# Novel quinazoline-1,2,3-triazole hybrids with anticancer and MET kinase targeting properties

**DOI:** 10.1038/s41598-023-41283-2

**Published:** 2023-09-06

**Authors:** Motahareh Mortazavi, Masoomeh Eskandari, Fatemeh Moosavi, Tahereh Damghani, Mehdi Khoshneviszadeh, Somayeh Pirhadi, Luciano Saso, Najmeh Edraki, Omidreza Firuzi

**Affiliations:** 1 Medicinal and Natural Products Chemistry Research Center, Shiraz University of Medical Sciences, Shiraz, Iran; 2 Department of Physiology and Pharmacology “Vittorio Erspamer”, Sapienza University of Rome, P.Le Aldo Moro 5, 00185 Rome, Italy

**Keywords:** Drug screening, Oncogenes, Cancer, Drug discovery, Virtual drug screening

## Abstract

Oncogenic activation of receptor tyrosine kinases (RTKs) such as MET is associated with cancer initiation and progression. We designed and synthesized a new series of quinazoline derivatives bearing 1,2,3-triazole moiety as targeted anticancer agents. The MET inhibitory effect of synthesized compounds was assessed by homogeneous time-resolved fluorescence (HTRF) assay and western blot analysis. Sulforhodamine B assay was conducted to examine the antiproliferative effects of synthetic compounds against 6 cancer cell lines from different origins including MET-dependent AsPC-1, EBC-1 and MKN-45 cells and also Mia-Paca-2, HT-29 and K562 cells. The growth inhibitory effect of compounds in a three-dimensional spheroid culture was examined by acid phosphatase (APH) assay, while apoptosis induction was evaluated by Annexin V/propidium iodide method. Compound **8c** bearing *p*-methyl benzyl moiety on the triazole ring exhibited the highest MET inhibitory capacity among tested agents that was further confirmed by western blot findings. Derivatives **8c** and **8h** exhibited considerable antiproliferative effects against all tested cell lines, with more inhibitory effects against MET-positive cells with IC_50_ values as low as 6.1 μM. These two agents also significantly suppressed cell growth in spheroid cultures and induced apoptosis in MET overexpressing AsPC-1 cells. Moreover, among a panel of 24 major oncogenic kinases, the PDGFRA kinase was identified as a target of **8c** and **8h** compounds. The docking study results of compounds **8c** and **8h** were in agreement with experimental findings. The results of the present study suggest that quinazoline derivatives bearing 1,2,3-triazole moiety may represent promising targeted anticancer agents.

## Introduction

Cancer is a major leading cause of death and morbidity across the world^1^ and more efficient therapeutic options are urgently needed for this deadly disease. The past two decades have witnessed a remarkable shift from conventional chemotherapy to targeted therapies which often offer better safety and efficacy profiles^2^.

Receptor tyrosine kinases (RTKs) such as MET (hepatocyte growth factor receptor or c-MET proto-oncogene), VEGFR (vascular endothelial growth factor receptor), EGFR (epidermal growth factor receptor), PDGFRs (platelet-derived growth factor receptors), etc. are key regulatory signaling proteins that their aberrant activation directs the development and progression of many types of cancer, a phenomenon that makes these receptors promising therapeutic targets^3^. In this context, numerous drugs, either approved or under investigation, have been developed to pharmacologically modulate the activity of these oncogenic RTKs^4^.

MET receptor is an extensively studied RTK that has received much attention as a proven therapeutic target in various malignancies^5^. The MET kinase is activated by its natural ligand, hepatocyte growth factor (HGF) or scatter factor (SF)^6^. Aberrant HGF/MET signaling pathway activation may occur as a consequence of protein overexpression, gene amplification or activating mutations, which is associated with the development and progression of many types of cancer, including lung, renal, gastrointestinal, thyroid, and breast cancers as well as glioblastoma among others^6, 7^. Until now, several MET small molecule tyrosine kinase inhibitors, including crizotinib, cabozantinib, capmatinib, and tepotinib, are approved for the management of certain types of cancers^8, 9^ and several others are being actively discovered and developed^10, 11^.

**Figure 1:**
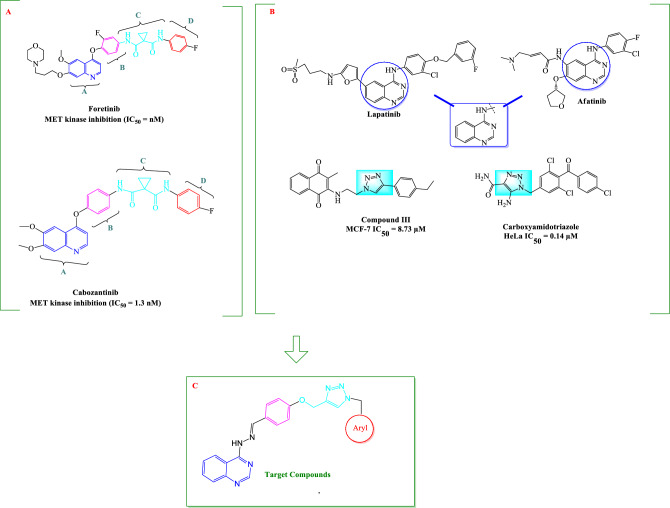
Design strategy for novel quinazoline-1,2,3-triazole hybrids compounds.

In the past few years, many researchers have demonstrated 4-aminoquinazoline derivatives as specific kinase inhibitors, including tyrosine kinase and serine/threonine kinases^12, 13^. A number of kinase inhibitor drugs with 4-aminoquinazoline core such as afatinib and lapatinib have been approved by Food and Drug Administration (FDA) for the treatment of several types of cancer (Fig. 1)^14–18^.

On the other hand, a series of 4-phenoxyquinoline derivatives have been reported as class II c-MET inhibitors, such as cabozantinib^19^, foretini^20^, kirin^21^, AM7^22^, and Amgen^23^ (Fig. 1). Among them, cabozantinib has been approved as a novel orally-available multi-kinase inhibitor for the treatment of patients with metastatic medullary thyroid cancer, renal cell carcinoma and hepatocellular carcinoma^24^. Foretinib which is currently undergoing phase III studies for different cancer types, is also a multi-kinase inhibitor targeting MET, VEGFR-2, RON and FLT1^25^.

Most type II MET kinase inhibitors share a common structure–activity relationship consisting of four distinct parts of A, B, C and D as shown in Fig. 1A. Moiety A is responsible for hydrogen bond formation with the backbone of MET kinase which is usually a nitrogen containing heterocyclic moiety such as quinoline, quinazolinone, and pyridine^26, 27^. On the other hand, parts B and D are usually a phenyl or substituted phenyl ring in the more promising compounds probably extended into the hydrophobic pocket formed by tyrosine residues to enhance the inhibitory activity^28^. The C fragment provides the five-atom linker with the capability of establishing H-bond interactions with the active site^26, 29^. In this work, 1,2,3-triazole fragment widely used as a building block in the design of anticancer agents (Fig. 1B) was employed as the C linker to establish the 5-atom linker and provide favorable H-bond interactions with the key residues of MET enzyme. Finally, different substituted benzyl derivatives and heteroaromatic pendants were applied as block D to provide the opportunity to investigate their influences on the anticancer as well as kinase inhibitory activity of designed compounds.

The compounds synthesized with the above-mentioned strategy were tested for MET kinase inhibitory activity in the enzyme-based and cell-based assays and also their antiproliferative effects against several solid tumors and leukemia cell lines, including MET-dependent cancer cells in two- and three-dimensional cell culture models. Compounds that showed the best results were selected for further studies including apoptosis induction effect, kinase selectivity profile and as well as molecular docking analysis.

## Results

### Chemistry of quinazoline derivatives

**Figure 2:**
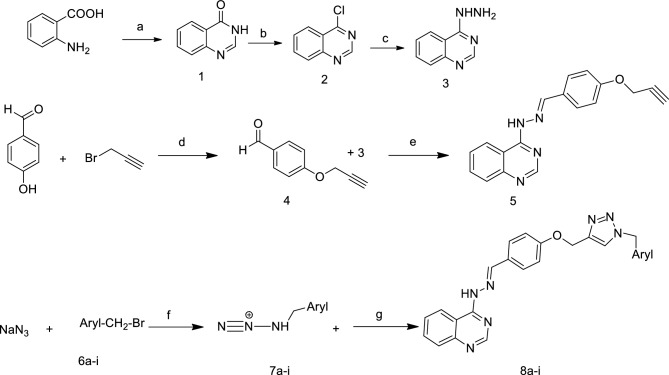
Schematic representation of the synthesis procedure of quinazoline derivatives (**8a**–**8i**). Reagents: (**a**) HCONH_2_, DMF, 24 h, 125–135 °C. (**b**) SOCl_2_, DMF, 80 °C, 5 h, (**c**) NH_2_NH_2_・H_2_O, EtOH, 3 h, reflux. (**d**) K_2_CO_3_, DMF, 80 °C, 18 h. (**e**) EtOH, 80 °C, Acetic acid 2 drops, 48 h (**f**) TEA, t-BuOH/water, 70 °C. (**g**) CuSO4・5H_2_O (5%), sodium ascorbate (15%), H_2_O, tButOH, 4–5 days, RT.

The summary of the synthetic route designed for the quinazoline derivatives are shown in Fig. 2.

Substituted 2-aminobenzoic acid, used as the starting material, was put to react with formamide in DMF at 125–135 °C to obtain the intermediate quinazolin-4-one (1). 4-Chloroquinazoline (2) was prepared by chlorination reaction with SOCl_2_. In the next step, reaction of compound 2 with hydrazine hydrate in isopropanol at 80 °C yielded quinazolin-4-yl-hydrazine. 4-(Prop-2-ynyloxy) benzaldehyde (4) was afforded via the reaction of 4-hydroxyaldehyde and 3-bromoprop-1-yne in the presence of K_2_CO_3_ in DMF at 80 °C. Further reaction of compound 3 and 4 in ethanol afforded 4-(2-(4-(prop-2-ynyloxy)benzylidene)hydrazinyl)quinazoline 5. Different halides of substituted benzyl and isoindoline derivatives were put to react with sodium azide in the presence of triethylamine in *t*BuOH and H_2_O at 70 °C. After around 45 min, intermediate compounds **(7a**–**i)** were formed and then catalytic amount of CuSO_4_·5H_2_O (5 mol %) and sodium ascorbate (15 mol %) were added. The resulting mixture was stirred at room temperature and completion of the reaction was monitored by using TLC to give compounds **8a**–**i**. The chemical structures of synthesized compounds are presented in Table 1.

**Table 1:** Chemical structures of the designed quinazoline derivative compounds.


Compound	Aryl
**8a**	
**8b**	
**8c**	
**8d**	
**8e**	
**8f**	
**8g**	
**8h**	
**8i**	

### MET kinase inhibitory effect

**Figure 3:**
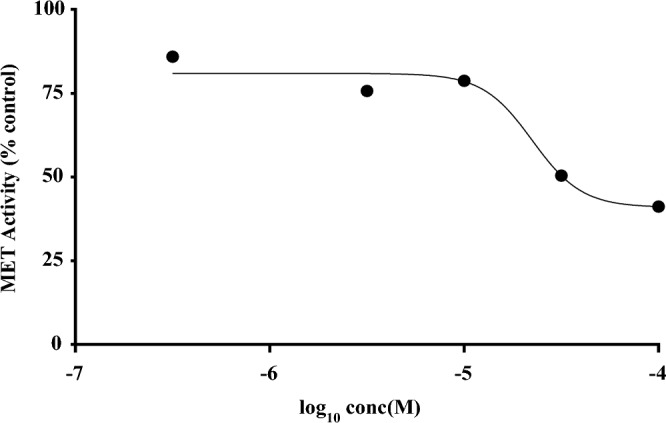
MET kinase inhibitory activity of **8c** determined by HTRF assay. Percent inhibition of MET kinase activity induced by compound **8c** at different concentrations was determined. The synthesized compound inhibited MET kinase activity with an IC_50_ value of 36.0 µM.

Synthesized compounds (**8a**–**i**) were examined for their MET kinase inhibitory activities using homogeneous time-resolved fluorescence (HTRF) assay by measuring the inhibition of phosphorylation of a substrate peptide. Only derivatives **8c** with *p*-methyl benzyl and **8h** with *p-t*butyl benzyl showed considerable inhibitory effects against MET kinase. The concentration-inhibitory effect curve of compound **8c** is shown in Fig. 3 (IC_50_ = 36.0 ± 9.5 µM). The second most potent compound, **8h**, showed inhibitory effects of 22.0 ± 7.4, 25.7 ± 4.7 and 32.9 ± 2.2 percent against MET kinase at concentrations of 10, 25 and 50 μM, respectively (mean ± S.E.M.). Crizotinib and cabozantinib were used as positive controls with IC_50_ values of 15.3 nM and 24.4 nM, respectively.

### Antiproliferative effects against cancer cells in monolayer culture

The antiproliferative effects of the synthetic compounds were evaluated by the sulforhodamine B (SRB) assay against seven different cancer cell lines, including AsPC-1 and Mia-Paca-2 pancreatic cancer, EBC-1 lung cancer with *MET* gene amplification, MKN-45 gastric cancer with *MET* gene amplification, HT-29 colorectal cancer cells as well as K562 leukemia cells. Cabozantinib and crizotinib were included as MET inhibitor reference compounds. Out of the 9 synthesized compounds (Table 2), derivatives **8c** and **8h** were the most effective ones at inhibiting the growth of the tested cancer cell lines, with IC_50_ values ranging from 6.1 to 34.4 µM. Notably, these two agents exhibited reasonable growth inhibitory effects against MET-positive tumor cells, including AsPC-1 cells with MET overexpression (IC_50_: 8c = 15.3, 8h = 9.7μM) and MET gene amplified cancer cells EBC-1 (IC_50_: 8c = 19.0 and 8h = 6.1 μM) and MKN-45 (IC_50_: 8c = 22.0, 8h = 12.0 μM)^30–34^. Altogether, it seems that alkyl derivatives of benzyl pendant could be superior anticancer agents comparing with other synthesized compounds. Moreover, considering the promising results of **8h** bearing *para*-tbutyl moiety on the benzyl pendant, bulkiness of substituted moiety would be favorable for the antiproliferative activity of compounds.

**Table 2:** Antiproliferative activity of synthetic quinazoline derivatives bearing various phenoxy-methylene-1,2,3-triazole pendants assessed by the SRB reduction assay.

Compound	AsPc-1	EBC-1	MKN-45	Mia-Paca-2	HT-29	K562
	IC_50_ (µM)					
**8a**	> 100	> 100	> 100	> 100	> 100	57.4 ± 21.9
**8b**	> 100	> 100	> 100	> 100	> 100	> 100
**8c**	15.3 ± 2.3	19.0 ± 0.7	22.0 ± 4.8	25.6 ± 2.4	21.0 ± 4.7	31.5 ± 6.4
**8d**	> 100	> 100	83.6 ± 6.0	> 100	93.4 ± 2.2	> 100
**8e**	> 100	> 100	> 100	> 100	> 100	61.5 ± 21.6
**8f**	> 100	> 100	56.6 ± 6.5	> 100	> 100	> 100
**8g**	> 100	> 100	67.7 ± 4.6	> 100	> 100	96.6 ± 1.1
**8h**	9.7 ± 1.9	6.1 ± 1.0	12.0 ± 3.9	11.5 ± 0.8	8.6 ± 1.3	34.4 ± 6.4
**8i**	> 100	89.4 ± 4.3	> 100	> 100	> 100	86.2 ± 3.0
Crizotinib	2.45 ± 1.3	0.006 ± 0.001	0.05 ± 0.003	2.36 ± 1.2	ND	ND
Cabozantinib	1.4 ± 0.1	0.059 ± 0.014	1.04 ± 0.06	3.8 ± 0.6	3.7 ± 0.67	4.0 ± 1.0

The results are expressed as mean ± S.EM.

### Inhibition of PDAC cell growth in a three-dimensional spheroid model

**Figure 4:**
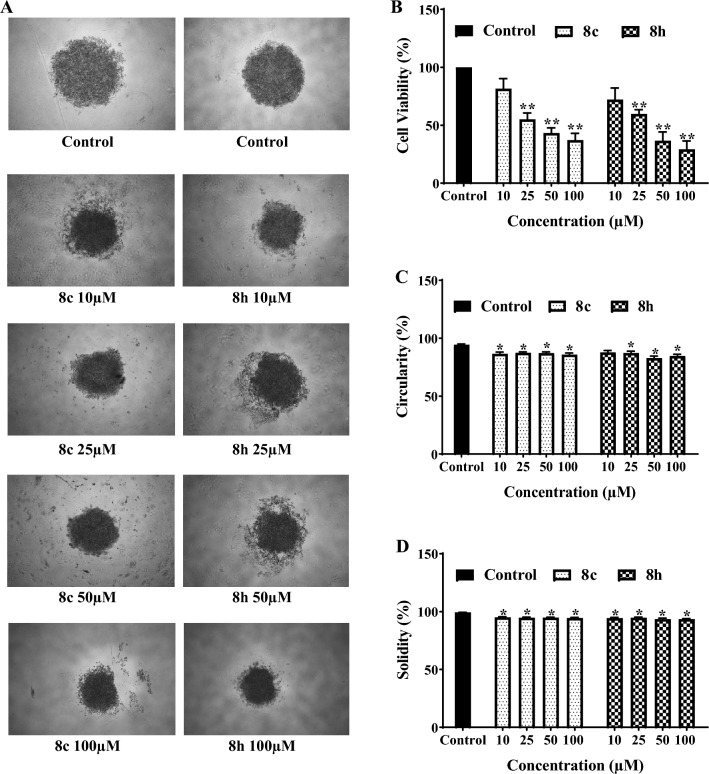
Inhibition of cancer cell growth in three-dimensional spheroid model. Spheroids of AsPC-1 cells were formed by liquid overlay technique in 96-well plates. (**A**) Representative images of spheroids treated with compounds **8c** and **8h** at concentrations of 10, 25, 50 and 100 μM are shown. (**B**) Growth inhibitory effects of compounds against AsPC-1 spheroids was measured by APH assay. (**C**) Circularity and (**D**) solidity of 3D spheroids after the administrations of synthesized compounds were measured by ImageJ software. Data are presented as mean ± S.E.M. of 3–6 separate experiments; The difference with control was statistically significant, at *(*p* < 0.05) and **(*p* < 0.01).

The growth inhibitory activity of the most potent antiproliferative agents in monolayer culture, **8c** and **8h,** was evaluated in a three-dimensional (3D) spheroid model. A single spheroid of MET-overexpressing AsPC-1 cell line was created in each well of 96-well plates with the liquid overlay technique. As shown in Figs. 4A,B, 8c,h inhibited the spheroid growth in a dose-dependent manner. Furthermore, the effect of these two agents on structural integrity parameters of spheroids was also quantified and a significant reduction in circulatory and solidity was observed after 72 h of treatment (Fig. 4C,D). It should be note that the IC_50_ values of compounds **8c** and **8h** in this assay (32.7 ± 7.2 µM and 36.6 ± 4.5 µM, respectively) were much higher than the same values obtained in the monolayer experiments (15.3 ± 2.3 and 9.7 ± 1.9 µM, respectively) showing that cancer cells are more resistant to anticancer agents when grow in 3D cultures^35^.

### Apoptosis induction in AsPC-1 cells

**Figure 5:**
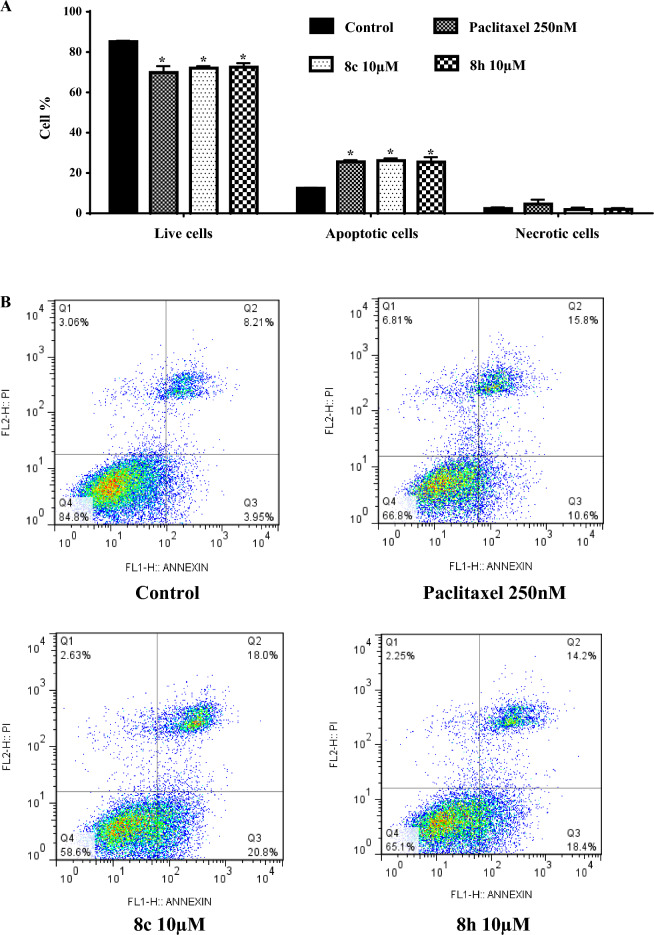
Apoptosis induction in AsPC-1 cells by synthesized derivatives **8c** and **8h**. AsPC-1 cells were treated with 10 µM of synthesized derivatives for 48 h. (**A**) Quantitative determination of apoptosis induced by synthesized derivatives with annexin V FITC/PI assay. Each bar represents the average percentage in each of the 4 quarters ± S.E.M from 3 replicates. Data are presented as mean ± S.E.M. *Denotes a statistically significant difference between drug-treated cells and untreated control (*p* < 0.05). (**B**) The apoptotic changes in the AsPC-1 cells were monitored by flow cytometry.

The apoptosis-inducing potential of the most effective antiproliferative compounds **8c** and **8h** was further investigated in the AsPC-1 cells using annexin V-FITC/PI assay. The analysis reported in Fig. 5 revealed that treatment with test compounds resulted in a significant and dose-dependent decline in the percentage of viable cells. Moreover, the total percentages of apoptotic cells in both early and late phases were found to be increased significantly in treated AsPC-1 cancer cells compared to untreated cells. The percentage of total apoptosis in the untreated cells was 12.4% (7.6% early and 4.8% late apoptosis), whereas this proportion increased to 26.1% in the cells treated with 10 μM **8c** (15.5% early and 10.7% late apoptosis) and 25.4% in the cells treated with 10 μM **8h** (15.8% early and 9.6% late apoptosis) (Fig. 5). Notably, necrotic cells showed no significant difference between treatment and control groups (Fig. 5).

### Inhibition of MET phosphorylation determined by western blot analysis

**Figure 6:**
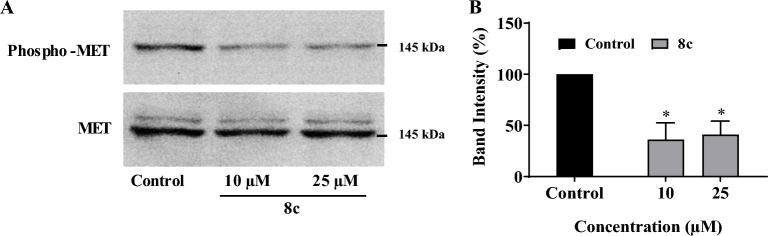
Effect of derivative **8c** on MET phosphorylation in AsPC-1 cancer cells. (**A**) AsPC-1 cells were seeded in 6-well plates and treated with different concentrations of **8c** for 3 h. Total cellular proteins were extracted and after separation by SDS-PAGE, blotted onto PVDF membranes and probed with specific anti-phospho-MET and anti-MET antibodies. A representative immunoblot is shown. (**B**) The inhibitory effect of **8c** on MET phosphorylation was quantified based on alterations of phospho-MET band intensities normalized to MET bands shown as percent inhibition compared to control untreated cells. Values represent the mean ± S.E.M. of 3–6 separate experiments. *The difference with control was statistically significant at *p* < 0.05. The blots were cropped to show only the MET and phospho-MET band areas pertaining to the studied compound. All replicates of the original blots are shown in the Supplemenary file S1.

Compound **8c** with the highest MET inhibitory capacity among the tested derivatives in the HTRF assay was further examined by immunoblotting and its inhibitory capacity on MET phosphorylation was analyzed in AsPC-1 cells. The compound was tested at two concentrations of 10 and 25 μM, and the results are shown in Fig. 6. As the blots demonstrate, treatment with this agent resulted in significant suppression of MET phosphorylation.

### Kinase selectivity profile

**Figure 7:**
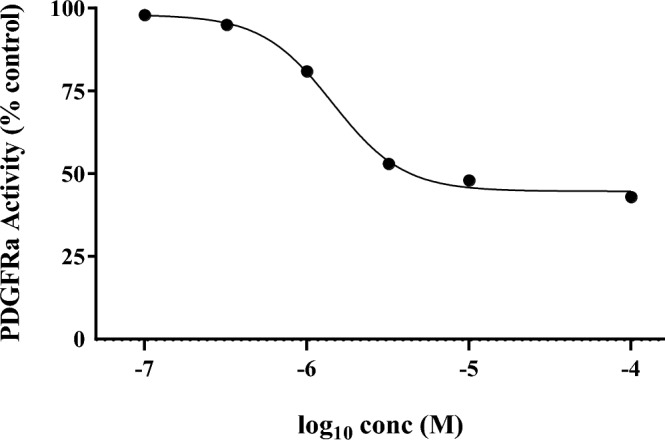
PDGFRA kinase inhibitory activity of compound **8c**. Percent inhibition of PDGFRA kinase activity caused by compound **8c** at different concentrations was determined. The synthesized compound inhibited PDGFRA activity with an IC_50_ value of 7.1 µM.

The most promising derivatives in this series, **8c** and **8h**, were further screened at the concentrations of 10 µM for their inhibitory activity against a panel of 24 other protein kinases, covering the well-known oncogenes across the human protein kinome using a radiometric assay. The compounds showed no inhibitory activities against most of the kinases present in this panel, indicating their relative selectivity (Table 3). However, both agents showed a considerable inhibitory potential against PDGFRA RTK. In particular, **8c** demonstrated the highest inhibitory effects of 58% at 10 μM. Therefore, the concentration-effect curve of this agent was also determined against PDGFRA protein, and the results are illustrated in Fig. 7.

**Table 3:** The inhibitory effects of synthesized derivatives 8c and 8 h against a panel of different protein kinases.

Kinase name	Kinase inhibition (%) at 10 μM	
	**8c**	**8h**
1. ABL (h)	8	17
2. ALK (h)	3	14
3. AKT1 (h)	15	26
4. AKT2 (h)	–	18
5. AXL (h)	12	− 1
6. CDK4/cyclinD3 (h)	2	–
7. EGFR (h)	8	− 5
8. FGFR1 (h)	− 11	− 14
9. FLT1 (h)	2	− 10
10. FLT3 (h)	8	18
11. FMS (h)	− 7	0
12. IGF-1R (h)	− 1	− 25
13. KDR (h)	− 5	− 2
14. KIT (h)	7	2
15. MAPK1 (h)	5	− 2
16. MAPK2 (m)	− 20	7
17. MEK1 (h)	–	− 10
18. MEK2 (h)	–	− 15
19. MTOR (h)	3	− 1
20. PDGFRA (h)	58	46
21. PDGFRB (h)	− 23	7
22. RET (h)	10	− 1
23. RON (h)	− 8	− 8
24. TRKA (h)	13	28

### In silico study

#### Molecular docking studies

**Figure 8:**
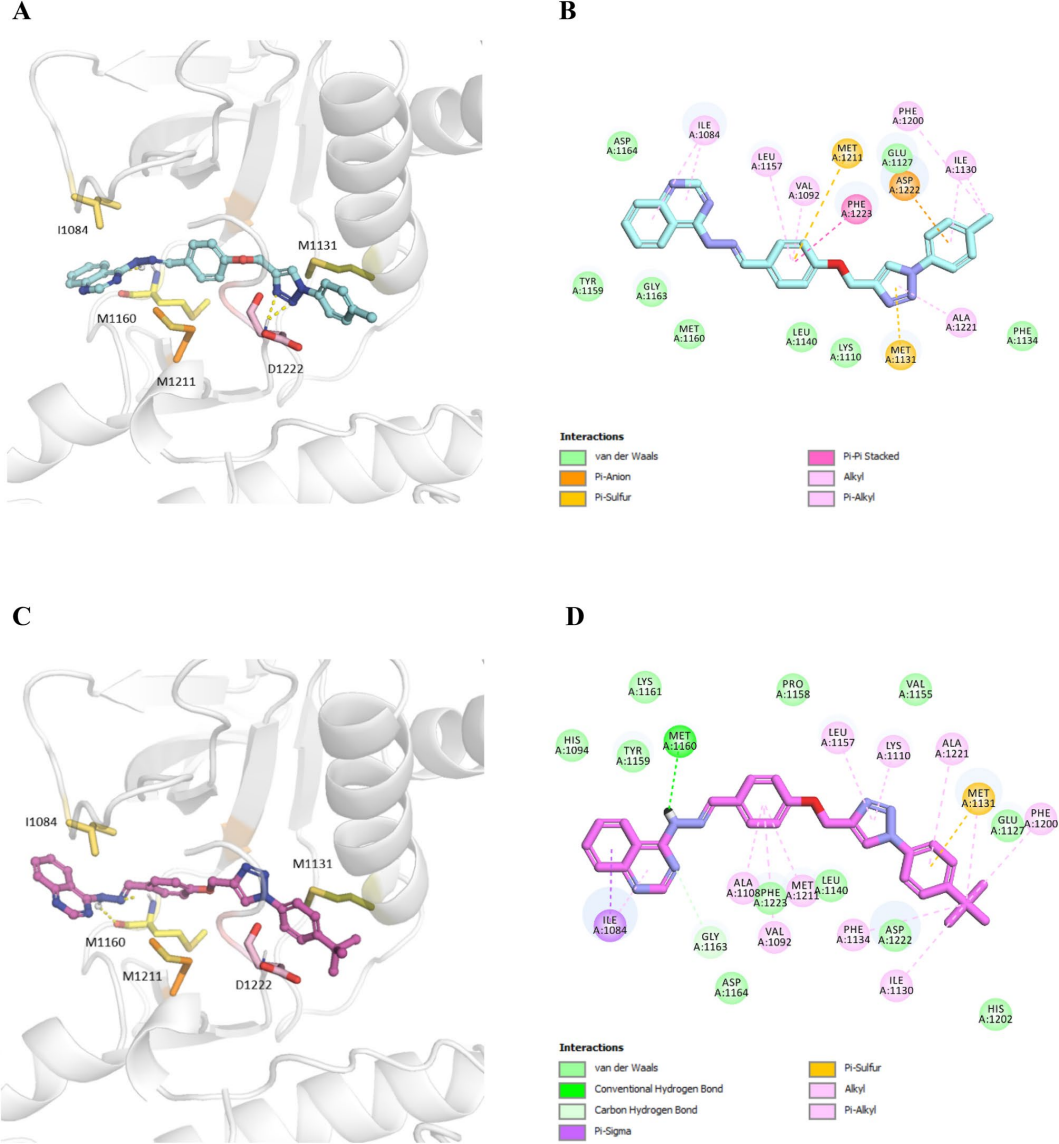
Molecular docking analysis of selected compounds against MET kinase. 3D interactions of **8c** (cyan) (**A**), 2D interactions of **8c** (**B**) 3D interactions of **8h** (pink) (**C**), and 2D interactions of **8h** (**D**) inside the MET active site are shown.

**Figure 9:**
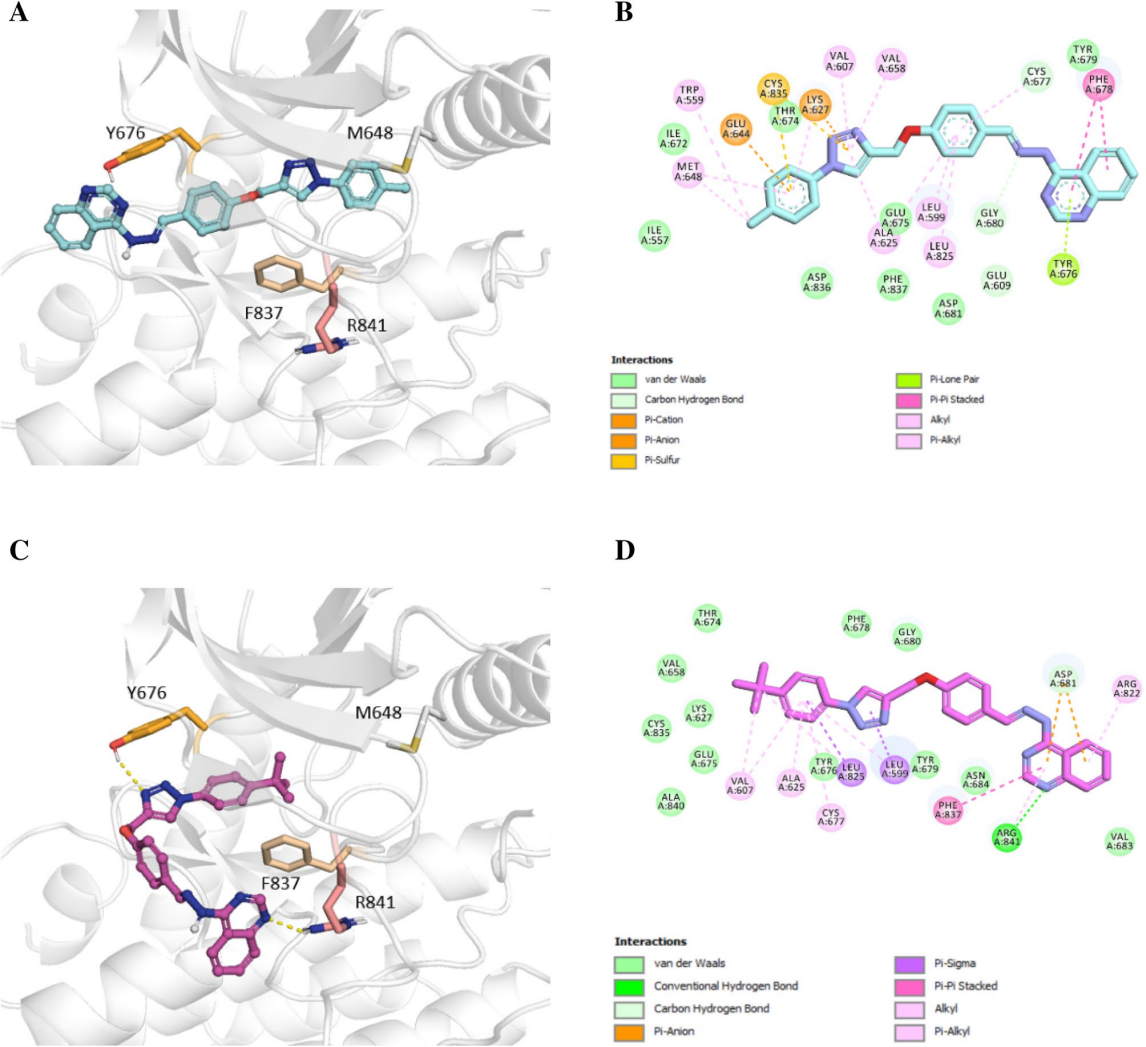
Molecular docking analysis of selected compounds against PDGFRA kinase. 3D interactions of **8c** (cyan) (**A**), 2D interactions of **8c** (**B**) 3D interactions of **8h** (pink) (**C**), and 2D interaction of **8h** (**D**) inside the PDGFRA active site are shown.

Molecular docking analysis was carried out in an attempt to evaluate the ability of synthesized compounds to interact with MET and PDGFRA kinases using smina docking. The human co-crystallized structure of MET (PDB code: 3LQ8) in complex with foretinib and PDGFRA (PDB code: 6JOK) in complex with sunitinib were utilized for the docking study. Figures 8 and 9 illustrates docking interactions of the most potent compounds **8c** and **8h** in the active site of MET and PDGFRA kinases, respectively. Minimized affinity energies and hydrogen bond interactions of all compounds against MET and PDGFRA kinases were shown in Table 4.

**Table 4:** Affinity of synthetic compounds for MET and PDGFRA kinases.

Compound	Affinity energy (kcal/mol)	
	MET	PDGFRA
Foretinib	− 11.05	− 6.86
Sunitinib	− 8.80	− 8.08
**8a**	− 9.94	− 7.81
**8b**	− 9.45	− 7.67
**8c**	− 10.88	− 7.89
**8d**	− 10.97	− 7.36
**8e**	− 9.91	− 7.64
**8f**	− 10.07	− 6.88
**8g**	− 10.13	− 7.62
**8h**	− 10.89	− 7.34

As shown in Fig. 8A–D, compounds **8c** and **8h** shared a similar binding mode inside the kinase domain of the MET active site, stretching from hinge region with quinazoline towards terminal helix. Compound **8c** made two hydrogen bond interactions with Asp1222. One from linker NH and one through the nitrogen atom of the quinazoline ring. Both compounds **8c** and **8h** made critical hydrogen bond interaction towards Met1160 via two N atoms of the triazole ring. This hydrogen bond interaction is also present in foretinib’s interaction with MET. In compound **8c**, the phenyl ring of the methoxyphenyl linker participated in the pi-pi stacked interactions with Phe1223. This phenyl ring made several pi-alkyl and alkyl interactions with residues around of **8c** and **8h** such as Val1092. Moreover, the terminal phenyl ring in compounds **8c** and **8h** formed pi-alkyl interactions with Ile1130 and Ala1221, respectively. In compound **8h**, triazole ring made pi-alkyl interactions with Leu1157, and Lys1110. The backbone structure of compound **8h** showed pi-alkyl interactions with Val1092, Met1211, Leu1157 and Ala1221.

On the other hand, examining **8c** binding mode with PDGFRA (Fig. 9A,B) showed that quinazoline ring participated in pi-pi stacked interaction with Phe678. Moreover, **8c** made pi-alkyl interactions with Met648, and Lys627 through the terminal methylbenzene group. Moreover, compound **8h** showed one hydrogen bond interaction with Arg841 from quinazoline ring and one from triazole ring towards Tyr676**.** Compound **8h** placed halfway through the hinge and terminal helix similar to sunitib. In addition, quinazoline ring participated in pi-pi stacked interaction with Phe837, the same as compound sunitinib.

## Discussion

The present study describes the synthesis of nine quinazoline derivatives bearing various phenoxy-methylene-1,2,3-triazole pendants and evaluation of their kinase inhibitory and anticancer properties. The findings showed that some of the compounds, in particular, **8c** bearing metyl phenyl pendant on the triazole ring, demonstrated considerable MET kinase inhibitory activity in cell-free HTRF as well as western blot assays. In parallel with these results, derivatives **8c** and **8h** exhibited remarkable growth inhibitory and apoptosis induction effects against different cancer cells, including MET-dependent cell lines in both monolayer and 3D spheroid models. Moreover, the kinase inhibitory potential assessment showed that compounds **8c** and **8h** might also suppress PDGFRA, another important oncogenic RTK. Computational studies supported our experimental observations and showed key interactions between synthesized derivatives and target kinases.

All the compounds were first screened for their MET kinase inhibitory potential using an HTRF assay. Among the tested compounds, only two derivatives **8c** and **8h** were active against the MET enzyme. In addition, **8c** with the highest MET inhibitory capacity significantly suppressed the phosphorylation levels of MET in western blot analysis. Activation of MET receptor begins with phosphorylation of tyrosine residues in the kinase domain, which is accompanied by the activation of downstream pathways leading to the emergence of diverse cellular hallmarks of cancer, including cell proliferation, survival, invasion and metastasis^6^. Therefore, these findings suggested high potential of these compounds in inhibition of important oncogenic pathways.

The antiproliferative effects of new derivatives were investigated against several cancer cell lines, including pancreatic cancer (AsPC-1, and Mia-Paca-2), lung (EBC-1), gastric (MKN-45) and colorectal cancer (HT-29) as well as leukemia cells (K562) in monolayer cell cultures. Among all synthesized compounds, **8c** and **8h** showed inhibitory effects on the proliferation of all tested cancer cells in monolayer culture. Interestingly, these two agents had the lowest IC_50_ values against MET-positive cells, including highly MET-expressing AsPC-1 cells as well as EBC-1 and MKN-45 cells harboring *MET* gene amplification. These cancer cells are dependent on MET signaling for proliferation and survival, and their growth is suppressed by MET inhibitors^30–34, 36^.

Three-dimensional cultures provide powerful tools offering reliable platforms that better represent physiological microenvironment in vivo, allowing cells to mimic real world features of solid tumors, such as gene expression patterns, microenvironment complexity, and drug resistance, among others^37^. In this context, the growth-inhibitory activities of the two promising compounds tested in 2D cell culture, **8c** and **8h,** were also examined towards MET-overexpressing AsPC-1 cells grown in 3D cultures. Based on our findings, both compounds significantly reduced the viability of AsPC-1 spheroids and also altered their physical properties, including circularity and solidity. Additionally, we observed that the 3D spheroid culture of AsPC-1 cells was more resistant to tested compounds compared to the same cells when grown in 2D cultures as IC_50_ values obtained for both compounds as well as cabozantinib (positive control) were elevated in comparison to monolayer cultures. This is consistent with the results observed in other studies^35, 37^ and could be explained by features such as a higher number of intercellular interactions and physical limitations, restricting the drug permeability in 3D compared to 2D cultures^38, 39^.

Moreover, apoptosis evaluation using flow cytometry with annexin V-FITC/PI staining revealed that incubation of AsPC-1 cells with **8c** and **8h** caused a significant increase in the number apoptotic cells. This capacity to induce apoptosis, but not necrosis, indicates high potential of these derivatives as anticancer agents. Dysregulation of the apoptotic signaling pathways is an important hallmark of cancer, contributing to tumor progression and drug resistance^40, 41^ and reactivation of this signaling network represents a rational strategy for developing more effective cancer therapeutics.^42^.

To investigate the inhibitory potentials of derivatives **8c** and **8h**, we evaluated the selected compounds in vitro against a panel of 24 well-known oncogenic kinases, mostly belonging to the RTK family. Based on kinase profiling results, both compounds exhibited negligible inhibitory activities against the majority of kinases tested; however, a considerable inhibitory effect of both agents, in particular, **8c,** was observed towards PDGFRA RTK. PDGFR kinase family consists of two receptors, PDGFRA and PDGFRB. In particular, it is well documented that PDGFRA signaling in malignant cells, and also tumor stroma and vasculature, is involved in the development and progression of several malignancies by promoting cell proliferation, migration and angiogenesis. Targeting the PDGFRA axis has been demonstrated to be an effective cancer therapeutic strategy^43–46^.

It is now well recognized that the majority of small molecule kinase inhibitors have great cross-reactivity within the kinase family due to the high structural homology among different kinases^3^. In our previous study, we also described the synthesis of a series of imidazopyridine hydrazone and quinazolinone hydrazide triazole derivatives as MET inhibitors and found they exhibited considerable potency against other kinases such as FLT3 and PDGFRA^35^ as well as ALK, AXL, FGFR1, FLT1 and FLT4 receptor tyrosine kinases^47^.

In summary, novel quinazoline derivatives bearing 1,2,3-triazole moiety seem to be promising antiproliferative agents with potential MET and PDGFRA inhibitory activities. Compounds **8c** and **8h,** bearing methyl and tert-buthyl phenyl rings, respectively, showed the highest inhibitory activities against the proliferation of various cancer cell lines. Both agents, in particular, **8c**, showed promising inhibitory effects against PDGFRA, in addition to MET receptor. The docking results were in parallel correlation with in vitro enzymatic assay of MET and PDGFRA.

## Materials and methods

### Chemistry of quinazoline derivatives

All reagents were purchased from the suppliers (Sigma-Aldrich, Fluka and Merck) without further purification. Reaction progress was observed by thin layer chromatography on glass-backed silica gel sheets (Silica Gel 60 GF254) and visualized under UV light (254 nm). Melting points were taken on Thermo Scientific Electrothermal digital apparatus (Thermo Fisher Scientific Inc.). ^1^H NMR (300 MHz) and ^13^C NMR (75 MHz) spectra were recorded on a Bruker AV300 (300 MHz) spectrometers at ambient temperature. FTNMR spectrometer is expressed in (δ, ppm) from the solvent resonance (Acetone, CDCl_3_, DMSO-d6 2.50 ppm). Mass spectra were obtained on Agilent Technology (HP) Model: 5973 Network Mass Selective Detector. The infrared (IR) spectra were run as KBr disk on Perkin- Elmer Spectrum RXI FTIR spectrophotometer.

### Synthesis of compounds 1–4

Compounds 1–4 were synthesized according to the reported procedures^48–50^ and confirmed by ^1^H NMR.

#### Synthesis of (E)-4-(2-(4-(prop-2-ynyloxy)benzylidene)hydrazinyl)quinazoline (5)

In a round-bottom flask a 10 ml ethanolic solution of 4 mmol of 4-(prop-2-ynyloxy) benzaldehyde (**4**) (MW = 160.17 g/mol) was prepared. 2 drops of acetic acid added, stirred at 80 °C for 1 h. The ethanolic solution of 4 mmol quinazoline-4-hydrazino (**3**), (MW = 160.18 g/mol) prepared and the two solutions were mixed and stirred at 80 °C. After 48 h the mixture was allowed to cool with ice bath, solid product thus separated was washed with ethanol, filtered and recrystalized from ethyl acetate to give pure compound **5.**

^1^H-NMR (300 MHz, CDCl_3_) *δ* = 2.59 (1H, s, –CH propargyl), 4.79 (2H, s, –CH_2_–O–), 7.08 (2H, d, J = 9 Hz, ArH), 7.56 (2H, d, J = 6 Hz, ArH), 7.79 (4H, m, ArH), 8.37 (1H, s, –CH=N–), 8.39 (1H, s, H-2), 9.56 ppm (1H, s, –NH–N=).

IR (KBr, cm^−1^): 3225 cm^−1^ (C(alkyne)–H stretching), 2112 cm^−1^ (monosubstituted alkyne C–C), 1672 cm^-1^ (C=N stretching), 1601 cm^−1^ (C=C aromatic stretch).

### Synthesis of target compounds (8a–i)

In a round-bottom flask a mixture of 0.9 mmol NaN_3_ (MW = 65 g/mol) and 1.1 mmol appropriate compound (**6a**–**i**, Fig. 2) in equal volume of water/^*t*^BuOH (total 5 ml) was stirred in the presence of triethylamine (Et_3_N, 1.3 mmol) for 45 min at room temperature which produces benzyl azide 7a-I^51^ and a solution of (E)-4-(2-(4-(prop-2-ynyloxy)benzylidene)hydrazinyl)quinazoline (**5**) (0.5 mmol), CuSO_4_. 5H_2_O (%5) (MW = 159.6 g/mol) and sodium ascorbate (%15) (MW = 198.1 g/mol) in equal volume of water/ ^t^BuOH were prepared. In second step of this stage the two solutions were mixed and stirring at room temperature for 4 days. After completion of the reaction, the mixture filtered, dried and crystallization of the residue from appropriate solvent afforded pure products (**8a**–**i**).

#### *2-(4-((1-(4-Bromobenzyl)-1H-1,2,3-triazol-4-yl)methoxy)benzylidene)-1-(quinazolin-4-yl)hydrazine(8a)*

White yellow solid, MP: 207.3 °C, ^1^H-NMR (300 MHz, CDCl_3_): *δ* = 3.66 (brs, 1H, –NH–), 5.19 (2H, s, CH_2_), 5.44 (2H, s, CH_2_–O), 7.01–6.98 (2H, d, *J* = 7.5 Hz ArH), 7.10–7.08 (2H, d, *J* = 7.2 Hz, ArH), 7.50–7.43 (4H, m, ArH), 7.76–7.70 (4H, m, ArH), 8.27 (1H, s, CH=N), 8.30 (1H, s, triazol ring), 9.43 (1H, s, quinazoline-H-2) ppm. ^13^C-NMR (75 MHz, CDCl_3_): *δc* = 53.64, 62.12, 115.20 (2C), 115.27, 122.70, 126.24, 127.14, 127.42, 127.64, 129.75 (2C), 130.51 (2C), 132.39, 133.36, 134.37, 136.35, 146.35, 146.58, 146.64, 159.34, 161.43, 162.74 ppm. MS (EI) *m*/*z* (%):516 ((M + 2) + , 0.17), 514 (M + , 0.17), 265 (2.7), 236 (7.3), 216 (2), 171 (58), 146 (100), 144 (7.6), 119 (22), 90 (30), 63 (19). Elemental analysis (C_25_H_20_BrN_7_O) Calculated: C(58.38%), H(3.92%), N(19.06%). Found: C(57.27%), H(3.45%), N(18.96%).

#### 2-(4-((1-(4-Fluorobenzyl)-1H-1,2,3-triazol-4-yl)methoxy)benzylidene)-1-(quinazolin-4-yl)hydrazine (8b)

White yellow solid, MP: 204.9 °C, ^1^H-NMR (300 MHz, CDCl_3_): *δ* = 3.77 (brs, 1H, –NH–), 5.27 (2H, s, –CH_2_–), 5.54 (2H, s, –CH_2_–O–), 7.06–7.09 (4H, Ar–H), 7.28 (2H, brt, Ar–H is overlapped by CDCl_3_), 7.58 (2H, s, Ar–H), 7.78–7.84 (4H, brt*, J* = 7.2 Hz, 10.8 Hz, ArH), 8.38 (2H, s, triazol-H and –CH=N–), 9.51 (1H, s, quinazoline-H-2) ppm. ^13^C-NMR (75 MHz, CDCl_3_): *δc* = 53.61, 62.14, 115.19, 116.08 (2C), 116.37 (2C), 126.26, 127.15, 127.39 (2C), 127.69 (2C), 129.99 (2C), 130.10 (3C), 130.49 (2C), 134.32 (2C), 159.33, 161.45, 162.69 ppm. MS (EI) *m/z* (%): 453 (M + , 0.2), 288 (48), 244 (13), 222 (6), 171 (48), 146 (100), 119 (31), 109 (37), 90 (41), 63 (33). Elemental analysis (C_25_H_20_FN_7_O) Calculated: C(66.11%), H(4.45%), N(21.62%). Found: C(65.38%), H(4.12%), N(21.78%).

#### 2-(4-((1-(4-Methylbenzyl)-1H-1,2,3-triazol-4-yl)methoxy)benzylidene)-1-(quinazolin-4-yl)hydrazine (8c)

MP: point is 194.7 °C, ^1^H-NMR (300 MHz, CDCl_3_): *δ* = 2. 37 (3H, s, Me), 4.14 (brs, 1H, –NH–), 5.27 (2H, s, –CH_2_–), 5.56 (2H, s, –CH_2_–O–), 7.01 (2H, m, Ar–H), 7.31–7.39 (4H, m, aromatic), 7.57 (2H, m, Ar–H), 7.81 (4H, t, *J* = 7.8, 10.2 Hz, Ar–H), 8.38 (2H, s, triazol-H and –CH=N–), 9.51 (1H, s, quinazoline-H-2) ppm. ^13^C-NMR 125 MHz, CDCl_3_): *δc* = 54.33, 60.38, 62.18, 115.20 (2C), 126.21, 127.12, 127.37, 127.65, 128.15 (2C), 128.22, 128.89, 129.19 (2C), 129.84, 130.48 (2C), 134.31, 134.36, 146.34 (2C), 146.64, 159.33, 161.49, 162.71 ppm. MS (EI) *m*/*z* (%): 449 (M + , 0.1), 190 (7.5), 172 (10), 162 (30), 158 (16), 146 (100), 122 (6.4), 119 (17), 109 (88), 91 (100), 65 (30). Elemental analysis (C_29_H_23_N_7_O) Calculated: C(69.47%), H(5.16%), N(21.81%). Found: C(70.07%), H(4.91%), N(21.47%).

#### 2-(4-((1-(3,4-Dichlorobenzyl)-1H-1,2,3-triazol-4-yl)methoxy)benzylidene)-1-(quinazolin-4-yl)hydrazine (8d)

White yellow solid, MP: 224.6 °C, ^1^H-NMR (300 MHz, CDCl_3_): *δ* = 4.15 (brs, 1H, –NH–), 5.30 (2H, s, –CH_2_–), 5.52 (2H, s, –CH_2_–O–), 7.14–7.07 (3H, brt, Ar–H), 7.62–7.40 (4H, m, Ar–H), 7.85–7.79 (4H, brt, Ar–H), 8.38 (2H, s, triazol-H and –CH=N–), 9.53 (1H, s, quinazoline- H-2) ppm. ^13^C-NMR (75 MHz, CDCl_3_): *δc* = 53.01, 62.12, 115.20 (2C), 126.34, 127.13, 127.24, 127.39, 127.67, 129.97 (2C), 130.50 (2C), 131.21 (2C), 133.35, 133.44, 134.33, 134.48, 146.33 (2C), 146.64, 159.35, 161.39, 162.63 ppm. MS (EI) ***m/z*** (%): 504 (M+, 0.4), 297 (5.4), 265 (3.2), 212 (21), 172 (7.5), 146 (100), 118 (23), 109 (10), 91 (23), 63 (15). Elemental analysis (C_25_H_19_Cl_2_N_7_O) Calculated: C(59.53%), H(3.80%), N(19.44%). Found: C(59.78%), H(3.04%), N(19.31%).

#### 2-(4-((1-(4-Chlorobenzyl)-1H-1,2,3-triazol-4-yl)methoxy)benzylidene)-1-(quinazolin-4-yl)hydrazine (8e)

Yellowish solid, ^1^H-NMR (300 MHz, CDCl_3_): *δ* = 4.67 (brs, 1H, –NH–), 5.19 (2H, s, –CH_2_–), 5.44 (2H, s, –CH_2_–O–), 6.56–7.16 (6H, m, Ar–H), 7.27–7.49 (2H, m, Ar–H), 7.61–7.74 (4H, brt, Ar–H), 8.29 (2H, s, triazol-H and –CH=N–), 9.43 (1H, s, quinazoline-H-2) ppm. ^13^C-NMR (75 MHz, CDCl_3_): *δc* = 53.57, 62.13, 114.61, 115.19 (2C), 126.26, 127.12, 127.39, 127.66, 129.42 (2C), 129.46 (2C), 130.49 (2C), 131.87, 132.86, 134.33, 134.99, 146.33 (2C), 146.64, 159.34, 161.43, 162.68 ppm. MS (EI) *m/z* (%): 303 (2), 264 (0.3), 161 (0.3), 156 (16), 146 (100), 128 (7.7), 118 (18), 103 (9.1), 90 (6.8), 76 (23), 63 (8.5).

#### 2-(4-((1-(4-Nitrobenzyl)-1H-1,2,3-triazol-4-yl)methoxy)benzylidene)-1-(quinazolin-4-yl)hydrazine (8f)

Orang solid, MP: 208.4 °C, ^1^H-NMR (300 MHz, CDCl_3_): *δ* = 4.84 (brs, 1H, –NH–), 5.32 (2H, s, –CH_2_–), 5.69 (2H, s, –CH_2_–O–), 7.08–7.16 (2H, m, Ar–H), 7.43–7.86 (8H, m, Ar–H), 8.25 (2H, s, Ar–H), 8.38 (2H, s, triazol-H and –CH=N–), 9.54 (1H, s, H-2) ppm.^13^C-NMR (75 MHz, CDCl_3_): *δc* = 53.26, 62.10, 114.49, 115.20 (2C), 116.28, 122.93, 124.39 (2C), 127.12, 127.40, 127.67, 128.03, 128.65 (2C), 130.50 (2C), 134.35, 134.80, 146.32 (2C, 146.64, 161.36, 161.16, 162.59 ppm. MS (EI) *m/z* (%): 265 (1.6), 189 (6.4), 178 (5.1), 171 (1.7), 146 (100), 132 (5.5), 125 (12), 119 (24), 103 (11), 90 (19), 76 (26), 63 (19), 50 (18). Elemental analysis (C_25_H_20_N_8_O_3_) Calculated: C(62.49.25%), H(4.20%), N(23.32%). Found: C(61.90%), H(4.31%), N(22.74%).

#### 2-(4-((1-Benzyl-1H-1,2,3-triazol-4-yl)methoxy)benzylidene)-1-(quinazolin-4-yl)hydrazine (8g)

Yellow solid, MP: 218.6 °C, ^1^H-NMR (300 MHz, CDCl_3_): *δ* = 5.19 (2H, s, –CH_2_–), 5.49 (2H, s, –CH_2_–O–), 7.02 (2H, s, Ar–H), 7.22–7.31 (5H, m, Ar–H overlap by CDCl_3_), 7.47 (2H, s, Ar–H), 7.74 (4H, s, Ar–H), 8.33 (2H, s, triazol-H and –CH=N–), 9.44 (1H, s, quinazoline H-2) ppm. ^13^C-NMR (75 MHz, CDCl_3_): *δc* = 54.37, 62.17, 115.21 (2C), 126.21, 127.14, 127.38, 127.68, 128.16 (2C), 128.90 (2C), 129.20 (2C), 130.48 (2C), 132.30, 134.31 (2C), 146.22, 146.45, 159.33, 161.49, 162.73 (2C) ppm. MS (EI) *m/z* (%): 436 (M +, 0.7), 265 (1.2), 172 (11), 146 (46), 144 (40), 118 (11), 104 (11), 91 (100), 76 (13), 65 (9.8), 50 (4.7). Elemental analysis (C_25_H_21_N_7_O) Calculated: C(68.95%), H(4.68%), N(22.51%). Found: C(68.79%), H(4.54%), N(22.04%).

#### 2-(4-((1-(4-Tert-butylbenzyl)-1H-1,2,3-triazol-4-yl)methoxy)benzylidene)-1-(quinazolin-4-yl)hydrazine (8h)

White solid, MP: 205.4 °C, ^1^H-NMR (300 MHz, CDCl_3_): *δ* = 1.32 (9H, s, C(CH_3_)_3_, 3.50 (brs, 1H, –NH–), 5.26 (2H, s, –CH_2_–), 5.53 (2H, s, –CH_2_–O–), 7.07–7.58 (10H, m, Ar–H overlap by CDCl_3_), 7.81–7.84 (4H, brt, Ar–H), 8.37 (2H, s, triazol-H and –CH=N–), 9.51 (1H, s, quinazoline H-2) ppm. ^13^C-NMR (75 MHz, CDCl_3_): *δc* = 31.25, 34.67, 54.03, 62.19, 115.20 (2C), 122.74, 122.90, 126.11 (2C), 126.18, 127.12, 127.37, 127.65, 127.99 (2C), 130.48 (2C), 131.32, 134.31, 143.79, 146.34, 146.63, 152.06, 159.33, 161.52, 162.71 ppm. MS (EI) *m/z* (%): 492 ((M + 1)+, 0.3), 200 (7.6), 172 (8), 146 (76), 144 (43), 132 (11), 118 (19), 91 (100), 65 (15), 57 (10), 50 (12). Elemental analysis: (C_29_H_29_N_7_O) Calculated: C(70.85%), H(5.95%), N(19.94%). Found: C(69.98%), H(5.59%), N(19.15%).

#### 2-((3-Propyl)isoindoline-1,3-dione)-1H-1,2,3-triazol-4-yl)methoxy)benzylidene)-1-(quinazolin-4-yl)hydrazine (8i)

Orange solid^, 1^H-NMR (300 MHz, CDCl_3_) *δ* = 2.34–2.27 (2H, m, –CH_2_–), 3.41 (s, 1H, –NH–), 3.72–3.67 (2H, t*, J* = 6.6 Hz, –CH_2_–), 4.40–4.35 (2H, t,* J* = 6.6 Hz, –CH_2_–), 5.21 (2H, s, CH_2_–O), 7.05–7.02 (2H, d*, J* = 9 Hz, Ar–H), 7.50–7.45 (1H, t, *J* = 8.1 Hz), 7.82–7.66 (9H, m, Ar–H), 8.27 (1H, s, CH=N), 8.29 (1H, s, triazol-H), 9.42 (1H, s, H-2) ppm. ^13^C-NMR (75 MHz, CDCl_3_): *δc* = 29.44, 34.96, 47.94, 62.09, 115.26 (2C), 123.45 (2C), 123.58, 126.13, 127.15, 127.39 (2C), 127.65, 130.52 (4C), 131.85, 134.28 (2C), 146.36 (2C), 146.61, 159.29, 161.54, 162.85, 168.37 (2C) ppm.

### MET kinase enzymatic assay

A homogeneous time-resolved fluorescence (HTRF) assay was done to determination of the MET kinase inhibitory activity of the synthesized derivatives^35^. HTRF assay detects the phosphorylation level of a biotinylated tyrosine kinase substrate peptide (TK substrate) by MET kinase. The HTRF® KinEASE™ TK kit and MET kinase were purchased from Cisbio and Millipore, respectively. The optimal conditions for enzyme, substrate, ATP concentrations, and enzymatic reaction times were set.

In order to prepare the test compounds, after dissolving in DMSO, they were diluted in the reaction buffer containing 50 mM HEPES pH 7.0, 0.1 mM sodium orthovanadate, 0.01% BSA, 0.02% NaN3, 10 mM MgCl2, 5 mM MnCl2, 2 mM DTT. Then, 4 μl of test compound at different concentrations (10, 25, 50 and 100 μM final concentrations) were loaded in a white 384-well plate (Cisbio Cat Number: 6007299) and afterwards, 2 μl of MET kinase (0.25 ng/μL) were added and incubated for 10 min. Then, to initiate the reaction, 2 μL of TK substrate (1 μM final concentration), and 2 μL ATP dissolved in kinase buffer (25 μM final concentration) were added consecutively. After the incubation of the reaction mixture for 50 min at room temperature, in the next step, the phosphorylated peptide substrate was detected by adding 10 μL of a detection solution containing 5 μL Eu3 + cryptate-conjugated antibody and 5 μL Steptavidin-XL665 (125 nM final concentration). Finally, the Time Resolved-Fluorescence Resonance Energy Transfer (TR-FRET) signal was measured after an hour of incubation at room temperature. The plates were read at excitation of 337 nm and dual emission of 665 and 620 nm using a Bio-Tek multimode plate reader (Model Cytation 3).

The following equations were used to determine the inhibition rate (%):

$${\text{Ratio}}_{{{665}/{62}0}} = {\text{ Emission}}_{{\text{665 nm}}} /{\text{ Emission}}_{{{62}0{\text{ nm}}}}$$

$$\Delta {\text{R }} = \, \left( {{\text{Ratio Sample}}_{{{665}/{62}0}} - {\text{ Ratio Background}}_{{{665}/{62}0}} } \right)*{1}00/{\text{ Ratio Background}}_{{{665}/{62}0}}$$

$${\text{Inhibition }}\left( \% \right) \, = \, \left( {\Delta {\text{R}}_{{{\text{Control}}}} - \, \Delta {\text{R}}_{{{\text{Sample}}}} } \right)*{1}00 \, / \, \Delta {\text{R}}_{{{\text{Control}}}}$$

Background samples contained all reagents except for the enzyme. Control wells contained the same amount of DMSO contained in the sample. The final concentration of DMSO did not exceed 2%.

### Cell culture

EBC-1 (human lung adenocarcinoma cells with *MET* gene amplification) and Mia-Paca-2 (human PDAC cells) were obtained from Japanese Collection of Research Bio Resources Cell Bank (JCRB). AsPC-1 (human PDAC), MKN-45 (human gastric adenocarcinoma cells with *MET* gene amplification), HT-29 (human colorectal adenocarcinoma), and K562 cells (human chronic myelogenous leukemia) were obtained from the Iranian Biological Resource Centre, Tehran, Iran. AsPC-1, EBC-1, MKN-45, and K562 cells were cultured in RPMI 1640 medium containing 10% heat-inactivated fetal bovine serum (FBS) and 1% penicillin/streptomycin. Mia-Paca-2 and HT-29 cells were grown in DMEM low glucose, containing 10% and 20% heat-inactivated FBS, resoectively and 100 U/ml penicillin/streptomycin. All cells were grown in monolayer cultures at 37 °C in a humidified incubator with 5% CO_2_.

### Assessment of the antiproliferative effects against cancer cells

Antiproliferative effects of synthetic compounds were evaluated by the SRB assay as described previously^52^. Cancer cells were trypsinized, homogenous cell suspensions were prepared and 100 µl of cell suspensions were cultured at the densities of 5 × 10^4^ cells/ml (for AsPC-1 and Mia-Paca-2), 4 × 10^4^ cells/m (for EBC-1 and MKN-45), 3 × 10^4^ cells/ml (for K562) and 1 × 10^4^ cells/ml (for HT-29) in 96-well flat-bottom plates. After 24 h of incubation to allow cells to attach and resume optimal growth, the synthetic compounds dissolved in DMSO and diluted in the growth medium were added. One hundred µl of synthesized derivatives were added at different concentrations in triplicate and incubated for an additional 72 h at 37 °C. The cells were then fixed by gentle addition of 50 μl cold trichloroacetic acid (TCA) 50% (w/v, 10% final concentration) and incubated for 60 min at 4 °C. Then, the supernatant was discarded, and the plates were washed four times with distilled water and air dried. Afterwards, 100 μl of SRB solution 0.04% (w/v) dissolved in 1% acetic acid was added to each well, and plates were incubated for 15 min at room temperature. At the next step, the unbound dye was removed by washing four times with 1% acetic acid and the plates were air dried. The bound stain was finally solubilized with 150 μl of 10 mM Tris base solution, and the absorbance was recorded at a wavelength of 540 nm with a Bio-Tek microplate reader (Model Synergy HTX).

### Measurement of the anticancer effect in three-dimensional spheroid assay

Three-dimensional spheroid cell cultures were produced based on the liquid overlay technique^35^. Agarose solution in RPMI (1.5% w/v) was prepared and sterilized in an autoclave. Then, each well of 96-well flat-bottom plates was coated with 50 µl of agarose solution and was left to solidify at room temperature for at least 2 h. One hundred twenty-five µl of a suspension of AsPC-1 cells in RPMI medium containing 10% FBS at a density of 2 × 10^5^ cells/ml was added to each well. Afterwards, the plates were centrifuged at 700*g* for 5 min and incubated under standard culture conditions. After 48 h of incubation, which allowed one spheroid to be formed in each well, 100 µl of the medium was removed, and spheroids were treated with synthesized derivatives diluted in a fresh medium containing 10% FBS for 72 h.

At the final step, cell viability was measured by the acid phosphatase (APH) assay, which is based on the hydrolysis of the p-nitrophenyl phosphate (pNPP) and its conversion to yellow p-nitrophenol by intracellular acid phosphatases present in viable cells. Briefly, 160 µl of the medium was removed and 200 µl of a solution containing 2 mg/ml pNPP dissolved in 0.1 M sodium acetate at pH 4.8 were added to each well and incubated for 120 min at 37 °C. Afterwards, 10 µl of NaOH 1 M was added to each well to stop the reaction, and the absorbance was recorded at 405 nm within 10 min by a Bio-Tek microplate reader (Model Synergy HTX). The images of spheroids were recorded by a bright field microscope (Nikon model DS-Ri2) and analyzed with Nikon NIS-Elements AR imaging software for Windows version 4.30.

### Assessment of apoptosis in AsPC-1 cells

The ability of apoptosis induction of synthetic compounds was evaluated by FACS analysis with Annexin V-FITC/propidiuim iodide (PI) staining kit (BD Pharmingen, San Diego, CA, USA). AsPC-1 cells were cultured in 6-well plates at a density of 1 × 10^5^ cells/ml. After 24 h, the synthesized derivatives at different concentrations were added and incubated for 48 h. After addition of trypsin to each well, the cells were harvested, transferred to 1.5 ml tubes and washed twice with PBS. At the final step, the cells were stained with Annexin V-FITC (5 µl) and PI (5 µl), followed by analysis by a FACS Calibur flow cytometer (Becton Dickinson, Mountain View, CA, USA). The effects of synthetic derivatives on the apoptosis rate were evaluated based on the fluorescence signal of 20,000 events.

### Measurement of MET phosphorylation in cancer cells by western blot analysis

The effect of synthetic compounds on MET phosphorylation in AsPC-1 cells was investigated by western blot analysis. At first, the AsPC-1 cells were seeded in 6-well plates at a density of 250,000 cells/ml and incubated at 37 °C for 24 h. The cultured cells were then treated with the synthetic compounds at different concentrations for 3 h. Then, the cells were harvested via addition of ice-cold RIPA lysis buffer (20 mM Tris base, 150 mM NaCl, 1% Np40, 1 mM EDTA, 5% sodium deoxycholate and 0.1% SDS, pH 8.0) containing 1 µM phenylmethylsulfonyl fluoride (PMSF; SigmaAldrich), 10 mM Na_4_O_7_P_2_ and 2 mM Na_3_VO_4_ and by use of scrapers. Also, PMSF and a protease inhibitor cocktail (Roche) were added to the extraction buffer to prevent the breakdown of the proteins. The cell lysates were vortex-mixed for 20 min and then centrifuged at 12,000*g* for 20 min at 4 °C. Supernatants were transferred into fresh tubes, and stored at − 20 °C until use. Protein contents of the cell extracts were measured by a bicinchoninic acid protein assay kit (Quanti-Pro BCA, Sigma–Aldrich, St. Louis, USA) using bovine serum albumin as the protein standard. Equal amounts of extracted protein were separated on 7.5% SDS–PAGE at 150 V in 1 h and then transferred onto the PVDF membrane. In order to block the nonspecific binding sites, the PVDF membranes were floated in 4% BSA dissolved in Tris buffer saline containing 0.1% Tween-20 (TBST) for 50 min at room temperature. Proteins were then detected by incubation with specific primary antibodies, rabbit monoclonal anti-p-MET (dilution 1:1000, catalogue number: 3126, Cell Signaling, Danvers, MA), and rabbit monoclonal anti-MET (dilution 1:1000, catalogue number: 4560, Cell Signaling, Danvers, MA) overnight at 4 °C. After incubation with secondary antibody (goat anti-rabbit horse radish peroxidase-conjugated IgG, Cell Signaling, Danvers, MA) for 1 h at room temperature, immune-reactive bands were visualized using enhanced chemiluminescence detection substrates (Thermo Fisher Scientific, Waltham, MA). Images were obtained with a G: Box Chemi-XR5 GeneSys image analyzer. The band intensities were calculated with the software Gene Tools (SyneGene, Cambridge, UK) for Windows.

### Kinase inhibition assays

To characterize the selectivity of synthesized compounds, their ability to inhibit a panel of 24 important oncogenic kinases was determined at 10 µM by using a radiometric assays at Km ATP concentrations for each kinase. Moreover, a dose–response analysis was performed at six concentrations to determine the activity of compound **8c** against PDGFRA kinase, and IC_50_ value was estimated by curve fitting. The tests were performed by Eurofins Discovery (https://www.eurofinsdiscovery.com).

### In silico study

#### Docking analysis

Molecular docking analysis was carried out against MET and PDGFRA to investigate the binding modes and the critical molecular interactions between the synthetic compounds and the binding site of the targets, using the smina molecular docking software^53^. smina was developed based off Auto-Dock Vina to improve the development of scoring functions and energy minimization. The process of preparing the protein structure involved adding hydrogens, removing water molecules, and native ligands, followed by assigning Kollmann charges to the receptors. The compounds were drawn using the Marvin Sketch tool (http://www.chemaxon.com). The energy levels of the synthetic compounds were optimized using the steepest descent algorithm, using Open Babel^54^. The enzyme's binding site for the docking process was determined automatically using the coordinates of co-crystallized native ligands foretinib and sunitinib with MET and PDGFRA kinases, respectively. Afterward, the program smina was utilized to identify the ligands binding modes and their interactions within the active site of the enzyme.

### Supplementary Information

Supplementary Information.

## Data Availability

The original contributions presented in the study are included in the article/Supplementary Materials S1, further inquiries can be directed to the corresponding authors.
