# Supplementary material for: Novel quinazoline-1,2,3-triazole hybrids with anticancer and MET kinase targeting properties
Source: Sci Rep. 2023 Sep 6;13:14685. doi: 10.1038/s41598-023-41283-2 (PMC10482942; doi:10.1038/s41598-023-41283-2)
Supplement: Supplementary file 1 — Supplementary Information. [file 41598_2023_41283_MOESM1_ESM.docx]

**Novel** **Quinazoline-1,2,3-triazole Hybrids with Anticancer and MET Kinase Targeting Properties**

Motahareh Mortazavi^a^, Masoomeh Eskandari^a^, Fatemeh Moosavi^a^, Tahereh Damghani^a^, Mehdi Khoshneviszadeh^a^, Somayeh Pirhadi^a^, Luciano Saso^b^, Najmeh Edraki^a*^, Omidreza Firuzi^a*^

^a^ Medicinal and Natural Products Chemistry Research Center, Shiraz University of Medical Sciences, Shiraz, Iran

^b^ Department of Physiology and Pharmacology "Vittorio Erspamer", Sapienza University of Rome, P.le Aldo Moro 5, 00185 Rome, Italy

**Corresponding authors:**

Omidreza Firuzi, MD PhD

Medicinal and Natural Products Chemistry Research Center

Shiraz University of Medical Sciences, Shiraz, Iran

Phone: (+98)-71-3230-3872

Email: [firuzio@sums.ac.ir](mailto:firuzio@sums.ac.ir)

Najmeh Edraki, PharmD, PhD

Medicinal and Natural Products Chemistry Research Center

Shiraz University of Medical Sciences

Shiraz, Iran

Phone: (+98)-71-3230-7869

Email: [edrakin@sums.ac.ir](mailto:edrakin@sums.ac.ir)

|  |  | | | | | | | | | | | |
| --- | --- | --- | --- | --- | --- | --- | --- | --- | --- | --- | --- | --- |
| **Bright field figure** | **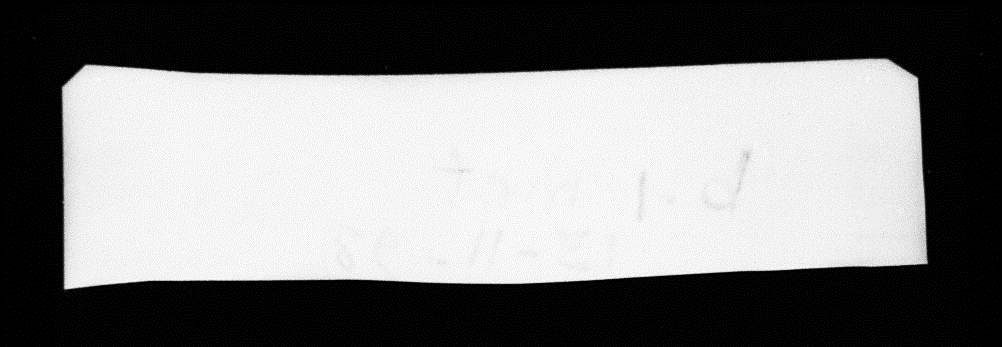** | | | | | | | | | | |  |
|  |  |  |  |  |  |  |  |  |  |  |  | **180 kDa**  **135 kDa**  **100 kDa**  **75 kDa** |
| **Phospho-MET** | **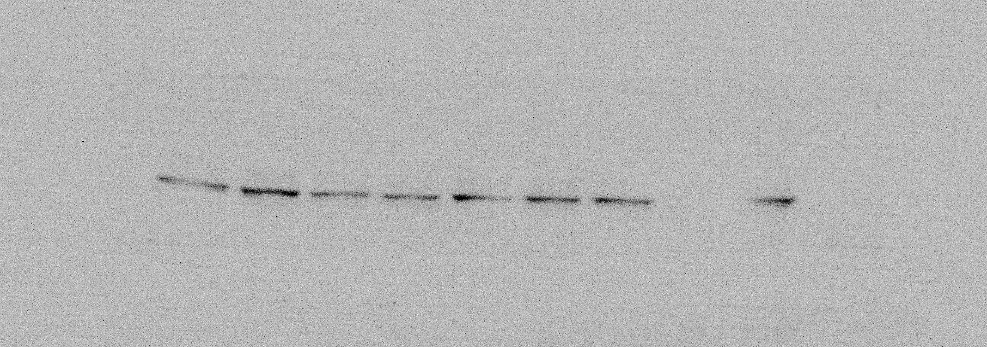** | | | | | | | | | | |  |
|  |  |  |  |  |  |  |  |  |  |  |  | **180 kDa**  **135 kDa**  **100 kDa**  **75 kDa** |
| **MET** | **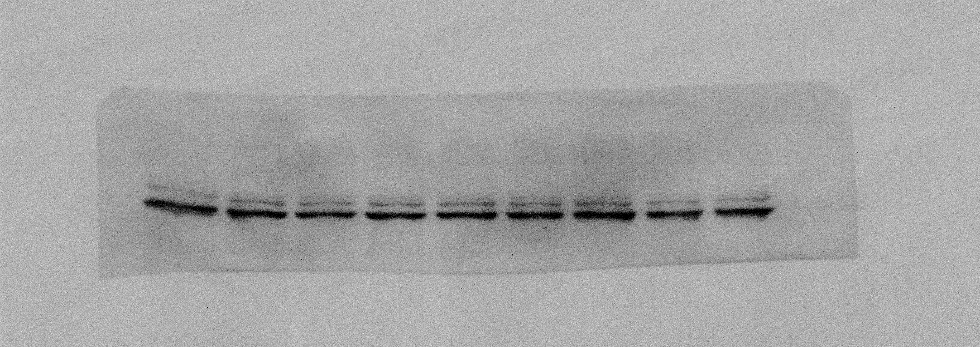** | | | | | | | | | | |  |
|  |  |  |  |  |  |  |  |  |  |  |  | **180 kDa**  **135 kDa**  **100 kDa**  **75 kDa** |
|  |  | **8c 5 µM** | **Control** | **8c 10 µM** | **8c 25 µM** |  |  |  | **Cabozantinib 200 nM** | **Control** |  | |

**Supplementary Figure 1a.** Western blot figure of experiment 1. The section enclosed in a box is presented in manuscript as Figure 6.

|  |  | | | | | | | | | | | |
| --- | --- | --- | --- | --- | --- | --- | --- | --- | --- | --- | --- | --- |
| **Bright field figure** | **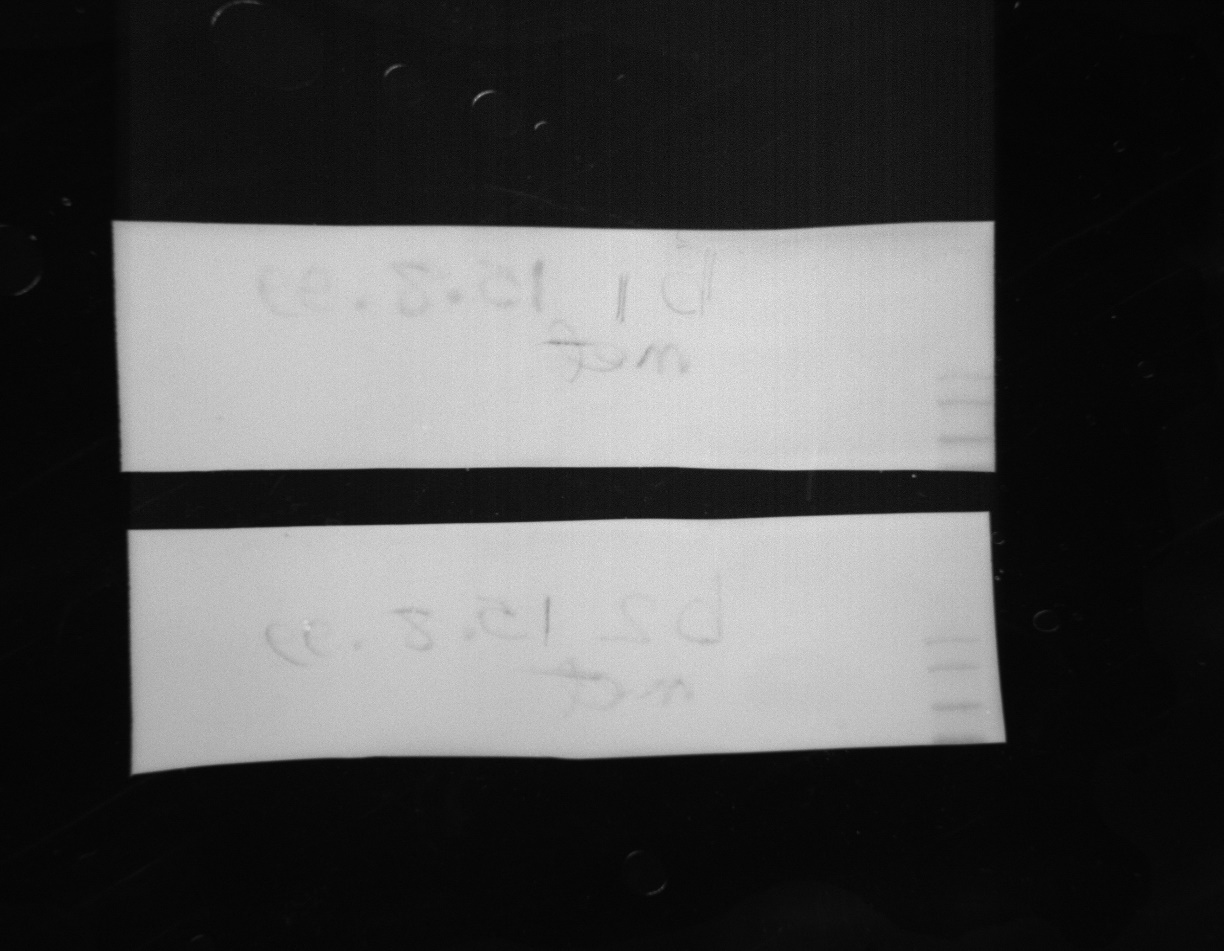** | | | | | | | | | | |  |
|  |  |  |  |  |  |  |  |  |  |  |  | **180 kDa**  **135 kDa**  **100 kDa**  **75 kDa** |
|  |  |  |  |  |  |  |  |  |  |  |  |  |
| **Phospho-MET** | **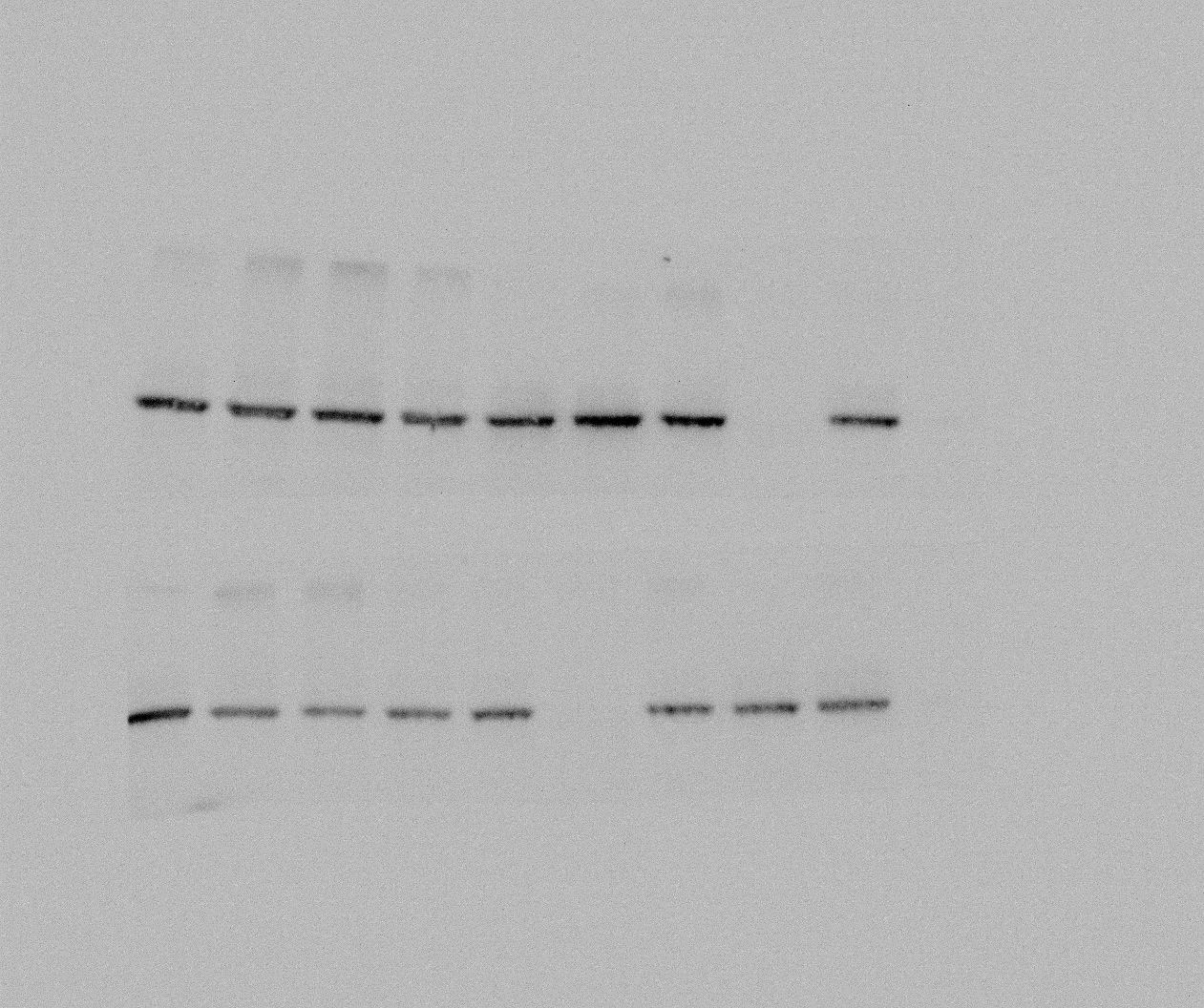** | | | | | | | | | | |  |
|  |  |  |  |  |  |  |  |  |  |  |  | **180 kDa**  **135 kDa**  **100 kDa**  **75 kDa** |
| **MET** | **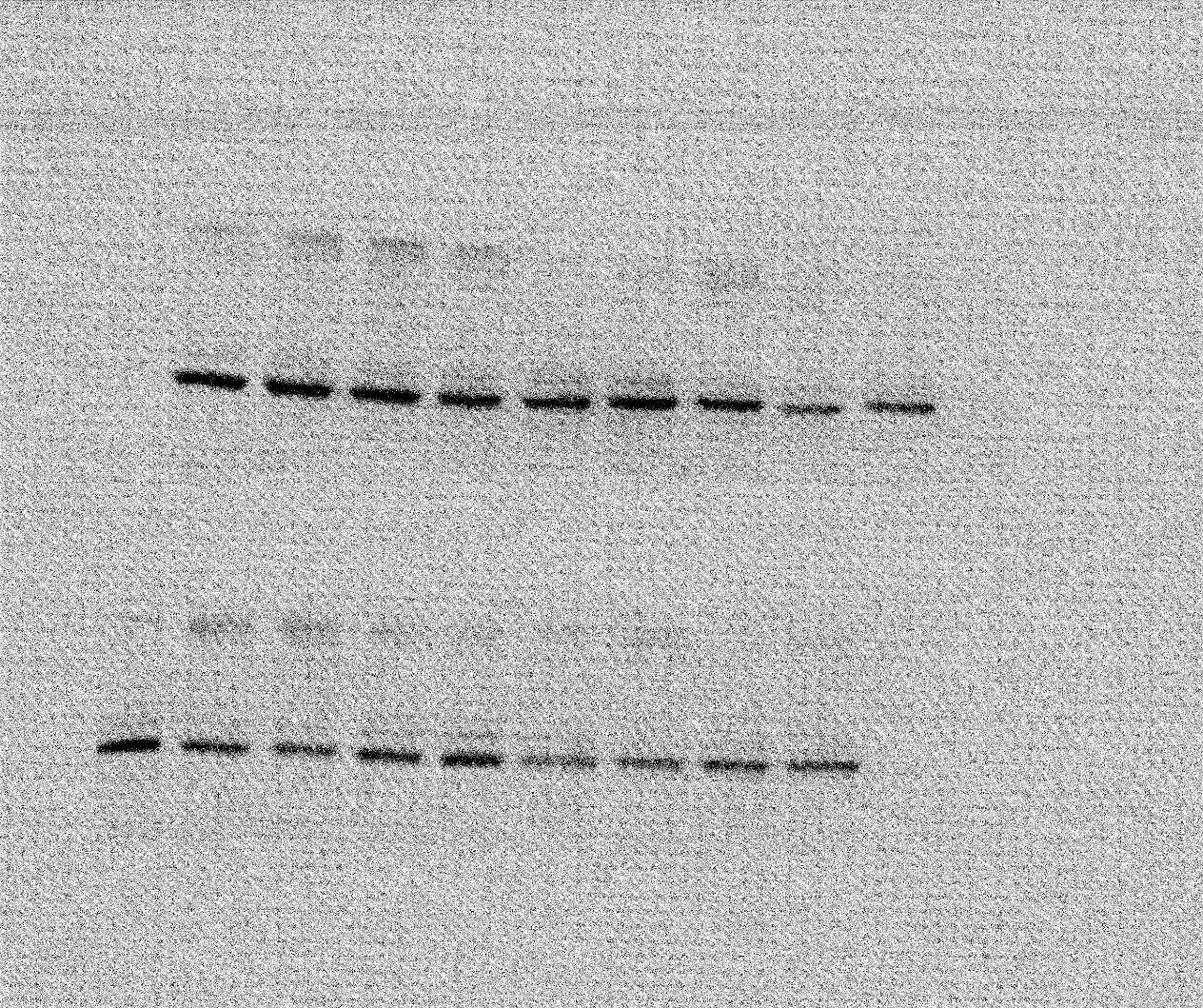** | | | | | | | | | | |  |
|  |  |  |  |  |  |  |  |  |  |  |  | **180 kDa**  **135 kDa**  **100 kDa**  **75 kDa** |
|  |  | **Control** | **8c 10 µM** | **8c 25 µM** |  |  | **Cabozantinib 200 nM** |  |  | **Control** |  | |

**Supplementary Figure 1b.** Western blot figure of experiment 2.

|  |  | | | | | | | | | | |
| --- | --- | --- | --- | --- | --- | --- | --- | --- | --- | --- | --- |
| **Bright field figure** | **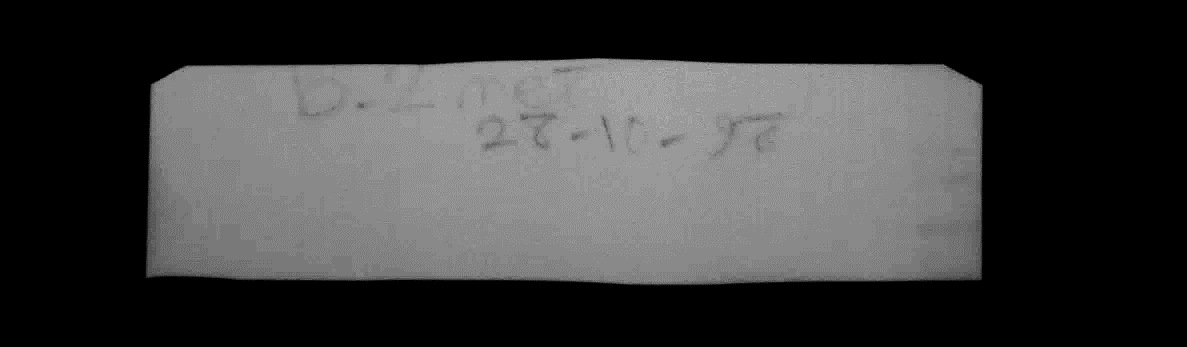** | | | | | | | | | |  |
|  |  |  |  |  |  |  |  |  |  |  | **180 kDa**  **135 kDa**  **100 kDa**  **75 kDa** |
| **Phospho-MET** | **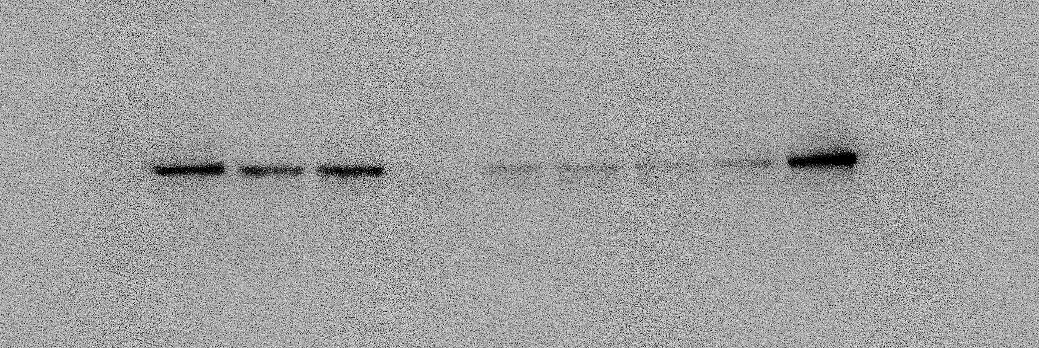** | | | | | | | | | | **180 kDa**  **135 kDa**  **100 kDa**  **75 kDa** |
|  |  |  |  |  |  |  |  |  |  |  |  |
| **MET** | **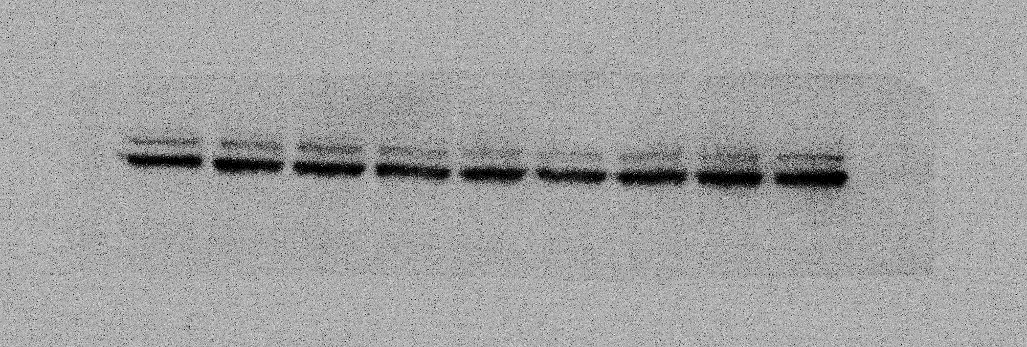** | | | | | | | | | |  |
|  |  |  |  |  |  |  |  |  |  |  | **180 kDa**  **135 kDa**  **100 kDa**  **75 kDa** |
|  |  | **Control** |  |  | **Cabozantinib 200 nM** |  |  | **8c 25 µM** | **8c 10 µM** | **Control** |  |

**Supplementary Figure 1c.** Western blot figure of experiment 3.

|  |  | | | | | | | | | | |
| --- | --- | --- | --- | --- | --- | --- | --- | --- | --- | --- | --- |
| **Bright field figure** | **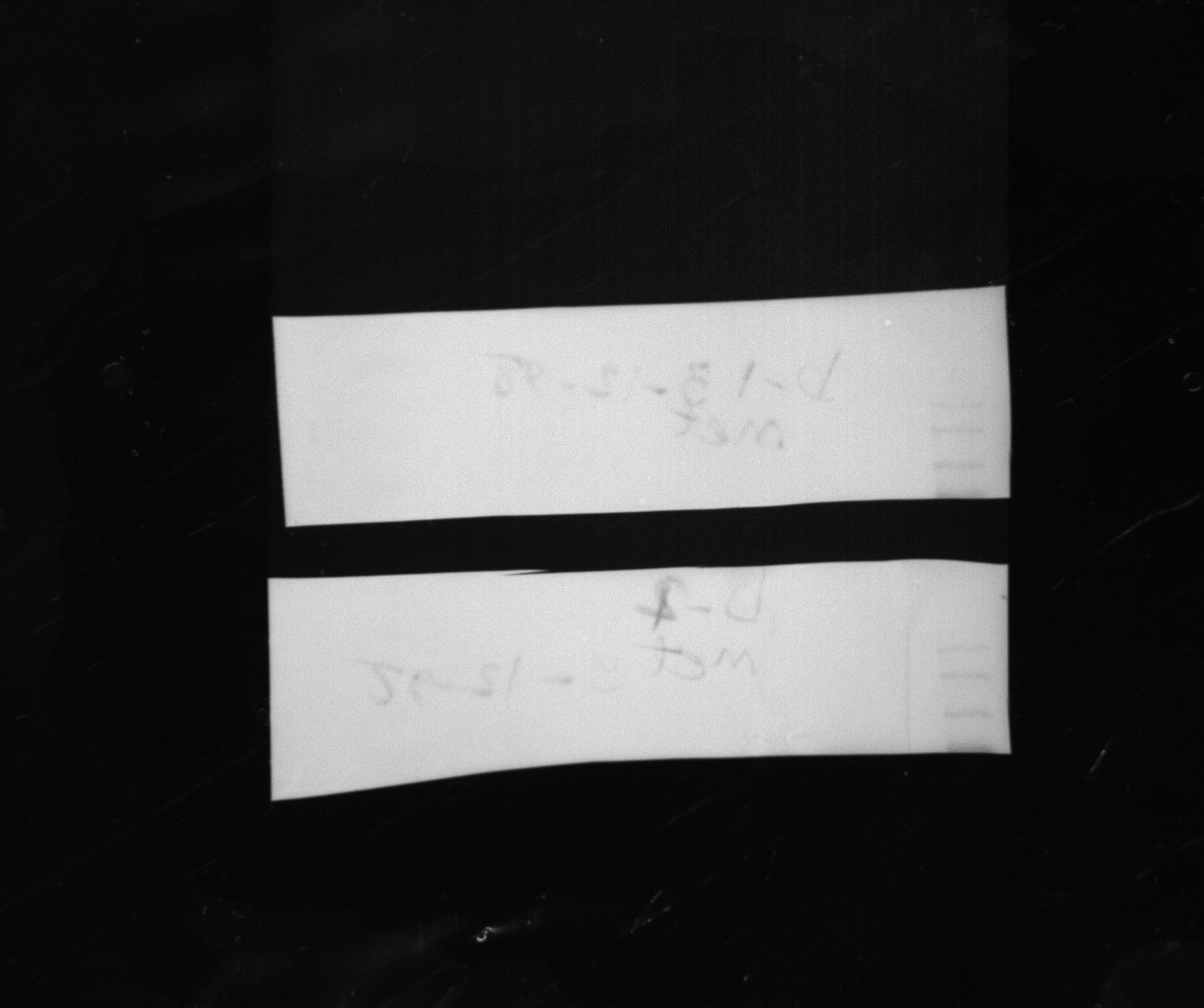** | | | | | | | | | |  |
|  |  |  |  |  |  |  |  |  |  |  | **180 kDa**  **135 kDa**  **100 kDa**  **75 kDa** |
| **Phospho-MET** | **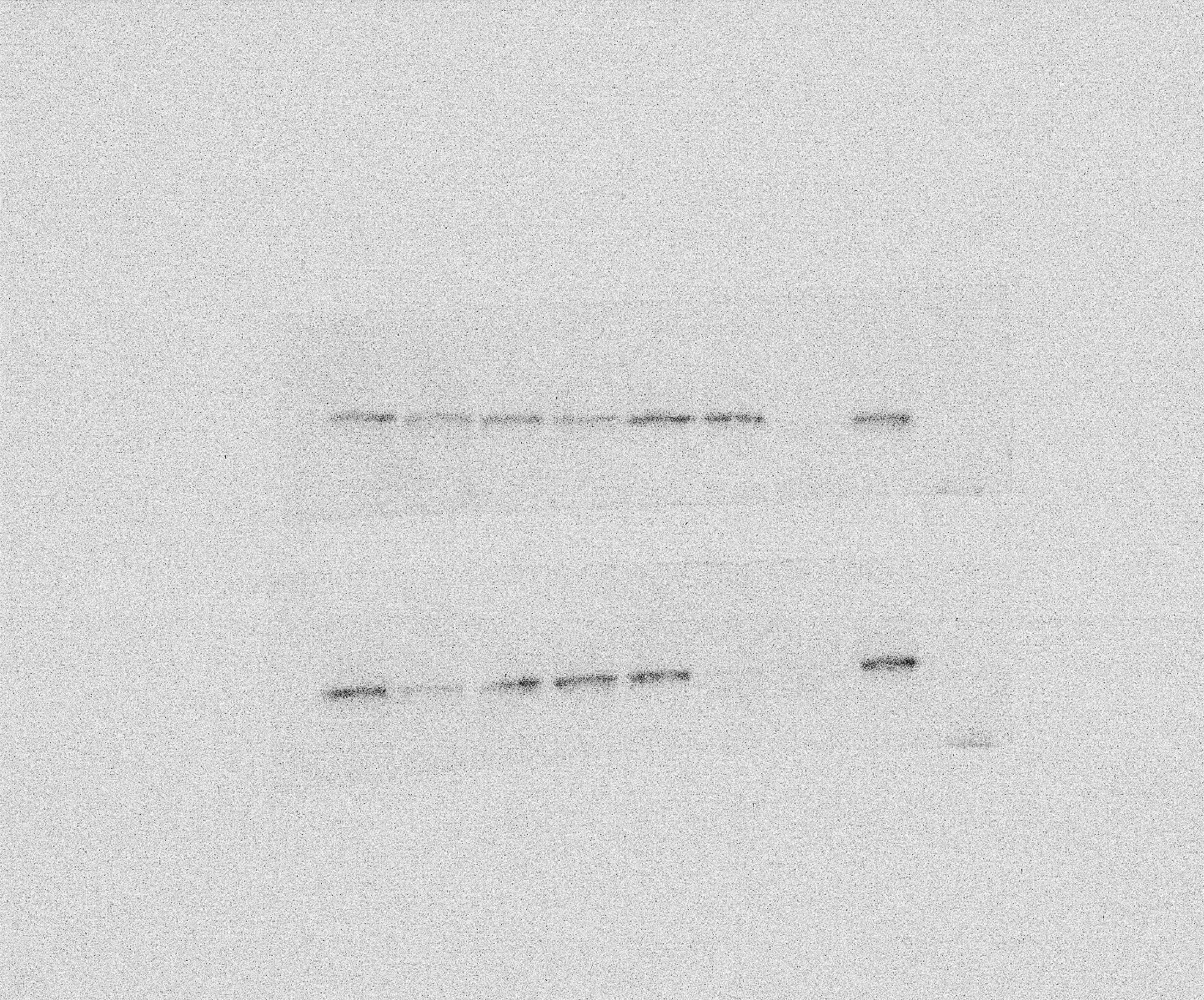** | | | | | | | | | |  |
|  |  |  |  |  |  |  |  |  |  |  | **180 kDa**  **135 kDa**  **100 kDa**  **75 kDa** |
| **MET** | **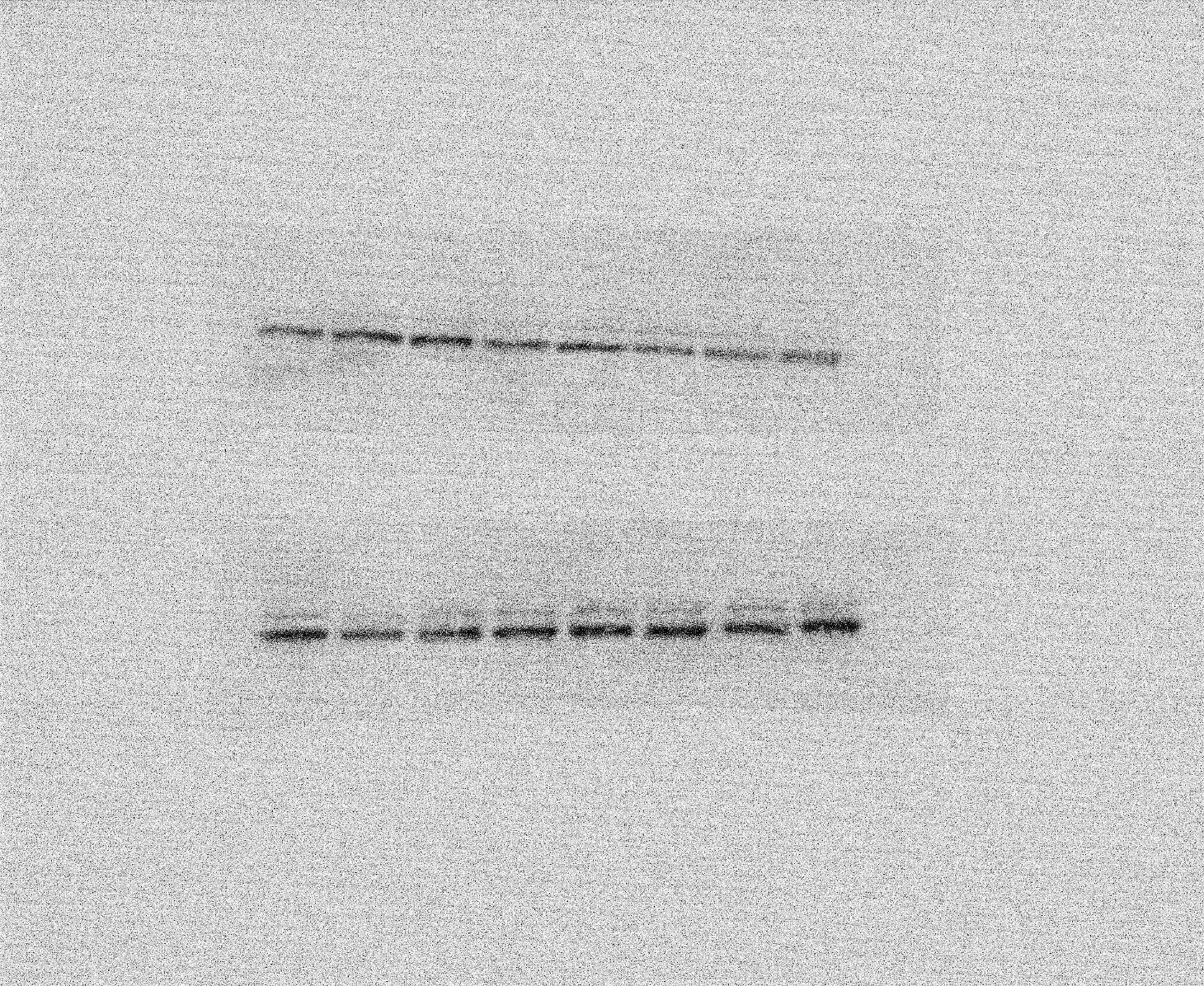** | | | | | | | | | |  |
|  |  |  |  |  |  |  |  |  |  |  | **180 kDa**  **135 kDa 100 kDa**  **75 kDa** |
|  |  | **Control** |  |  |  | **8c 10 µM** | **8c 25 µM** | **Cabozantinib 200 nM** | **Control** |  | |

**Supplementary Figure 1d.** Western blot figure of experiment 4.

| 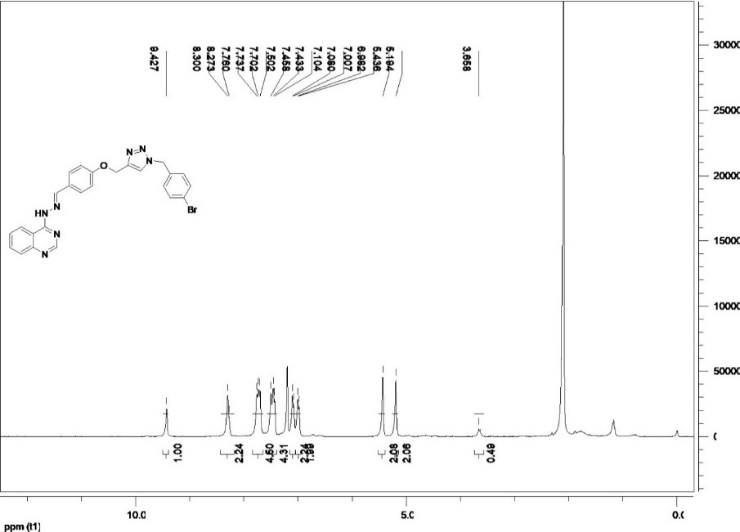 | 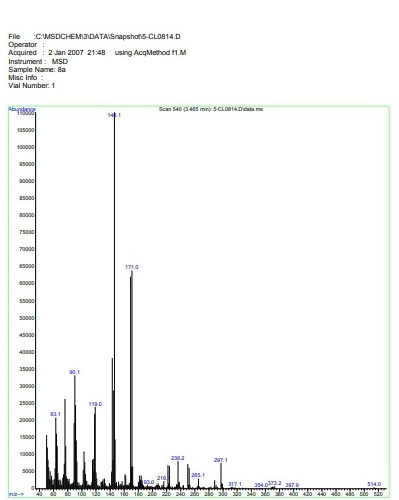 |
| --- | --- |
| 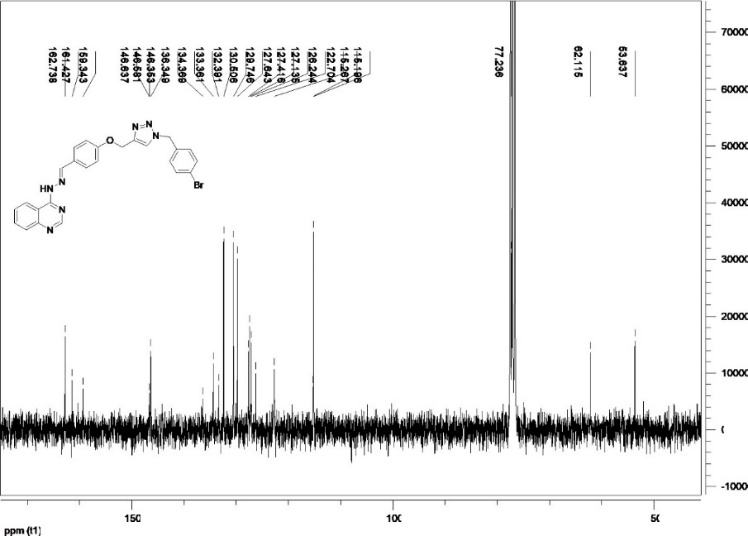 |  |
| **Supplementary Figure 2.** Chemical characterization of compound **8a.** | |

| 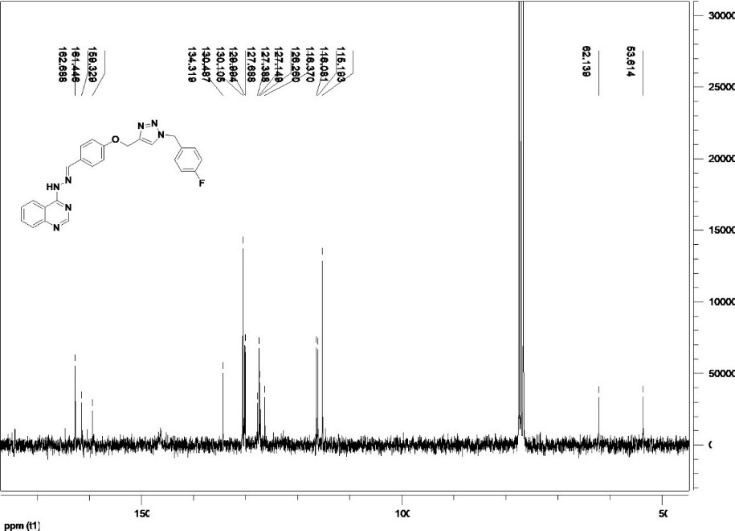 | 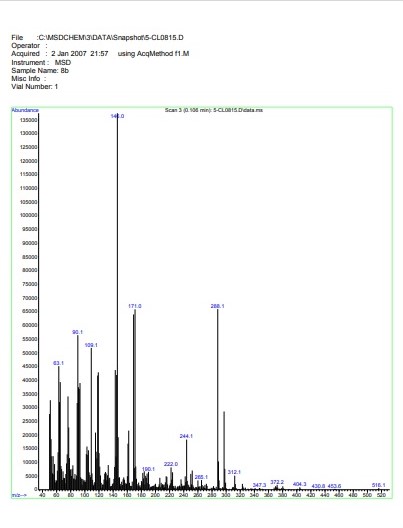 |
| --- | --- |
| 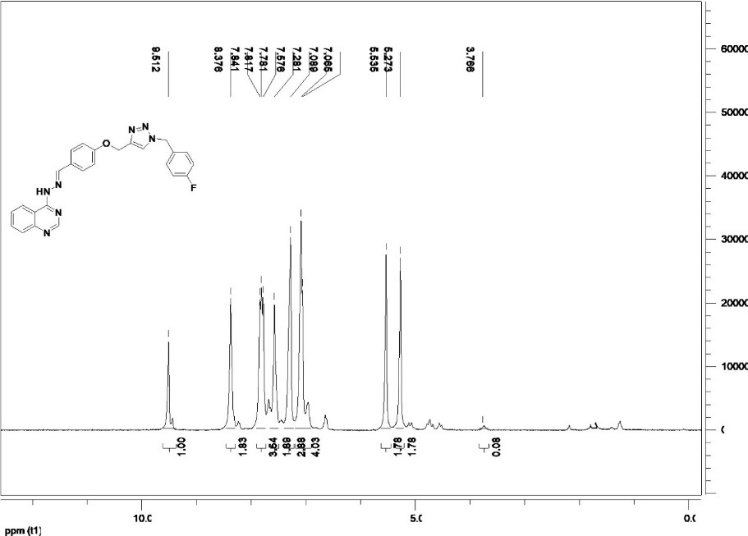 |  |
| **Supplementary Figure 3.** Chemical characterization of compound **8b.** | |

.

|  | 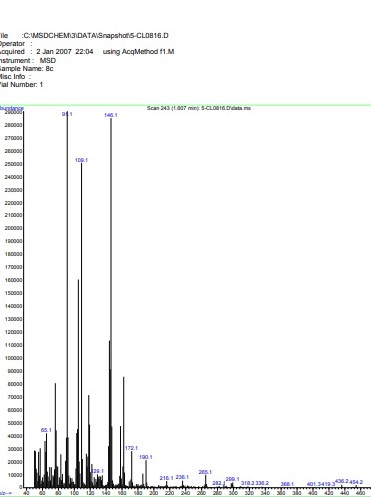 |
| --- | --- |
| 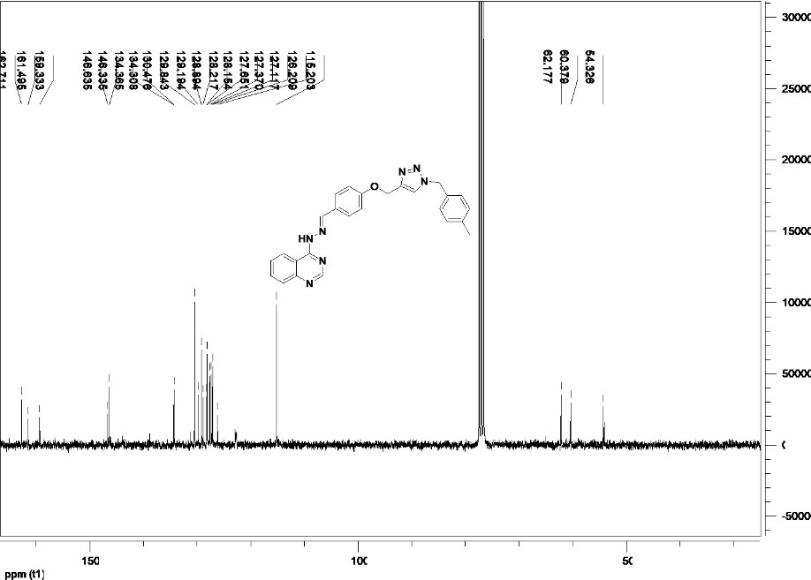 |  |
| **Supplementary Figure 4.** Chemical characterization of compound **8c.** | |

| 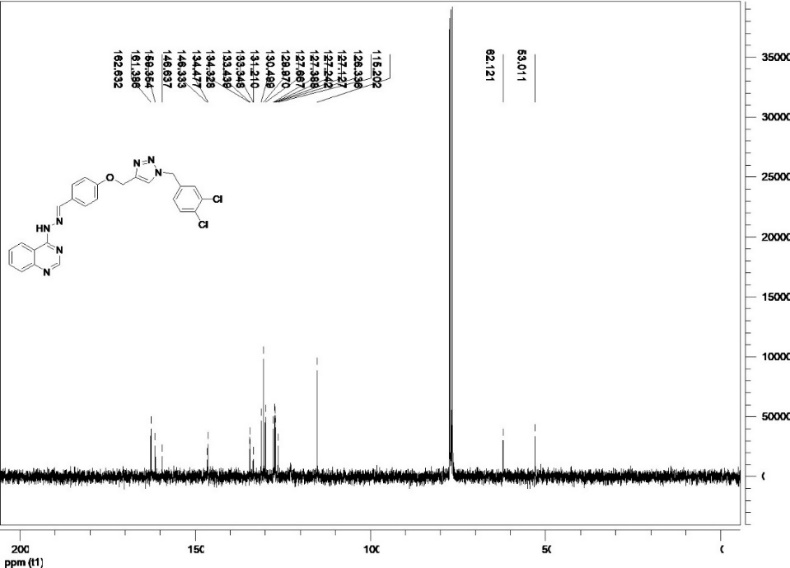 | 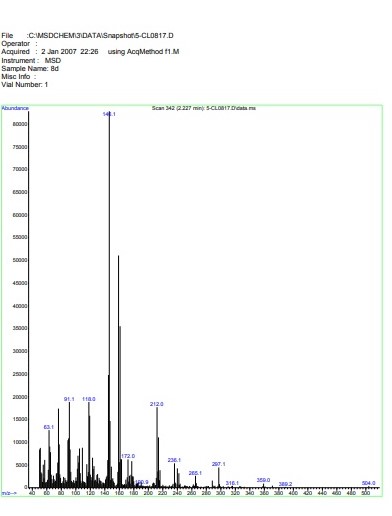 |
| --- | --- |
| 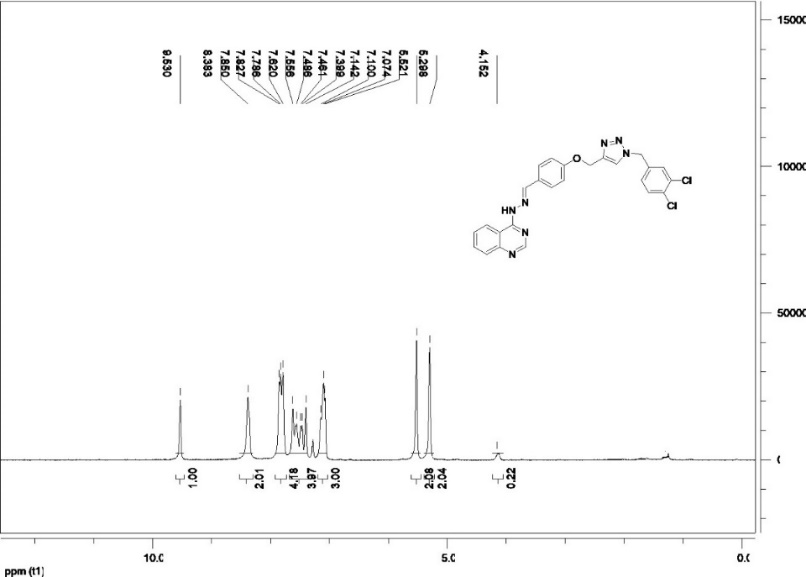 |  |
| **Supplementary Figure 5.** Chemical characterization of compound **8d.** | |

| 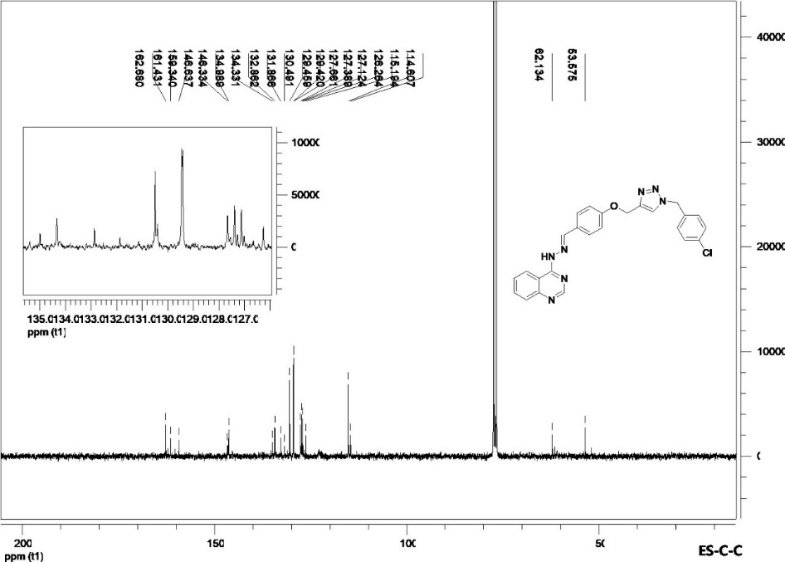 | 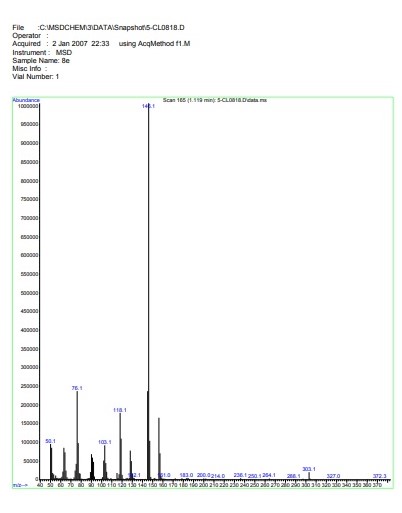 |
| --- | --- |
| 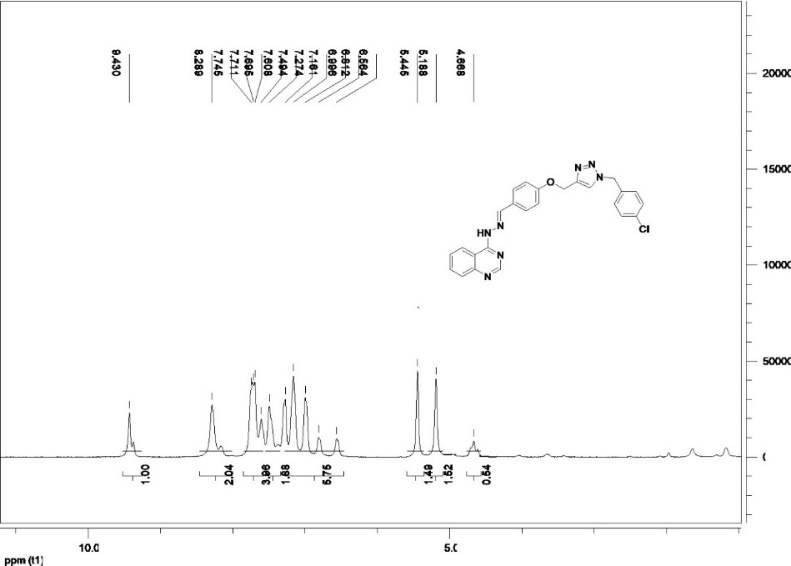 |  |
| **Supplementary Figure 6.** Chemical characterization of compound **8e.** | |

| 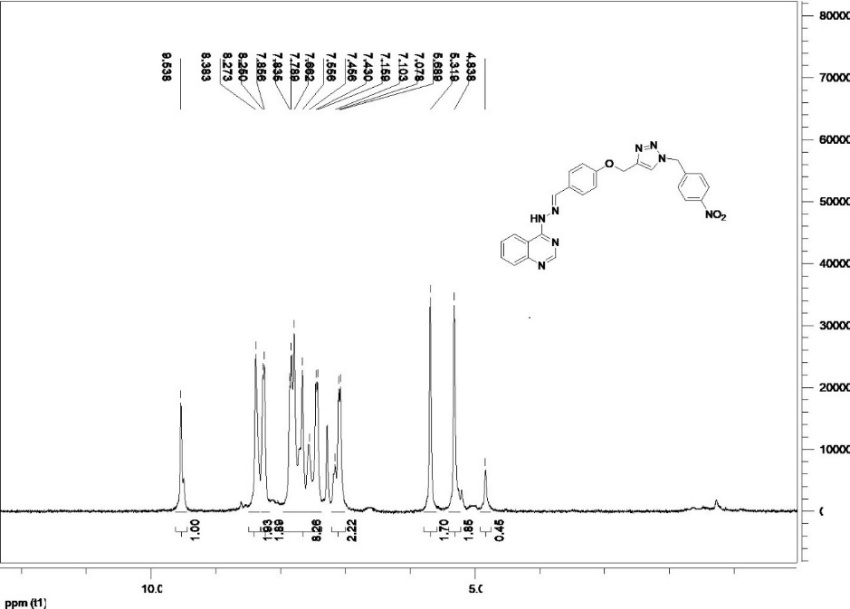 | 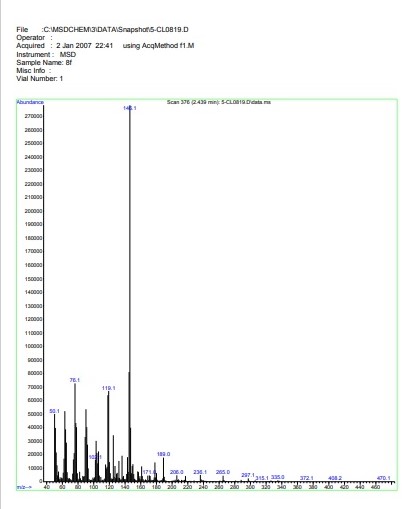 |
| --- | --- |
| 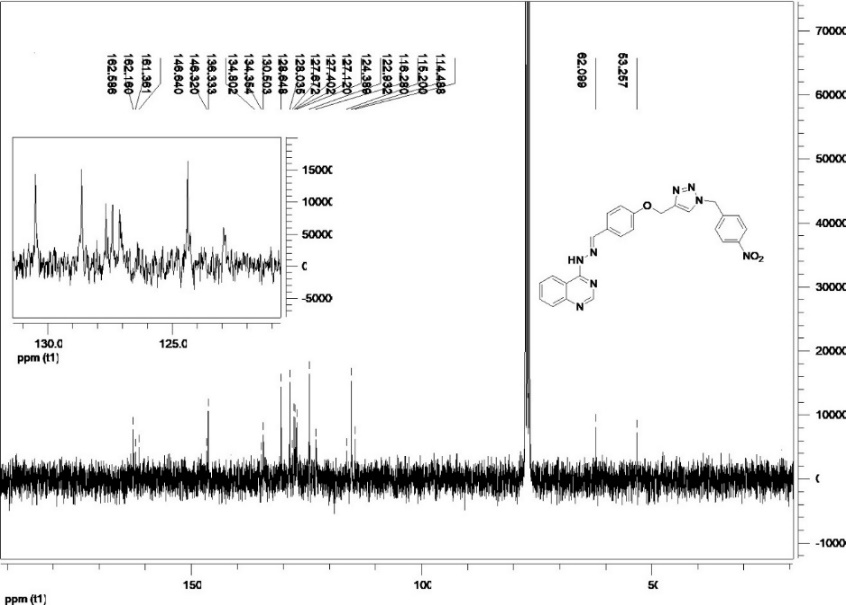 |  |
| **Supplementary Figure 7.** Chemical characterization of compound **8f.** | |

| 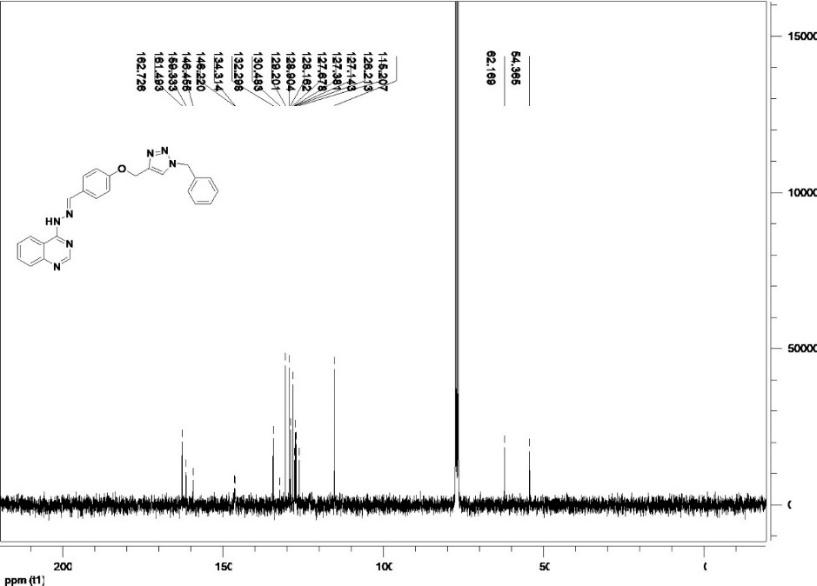 | 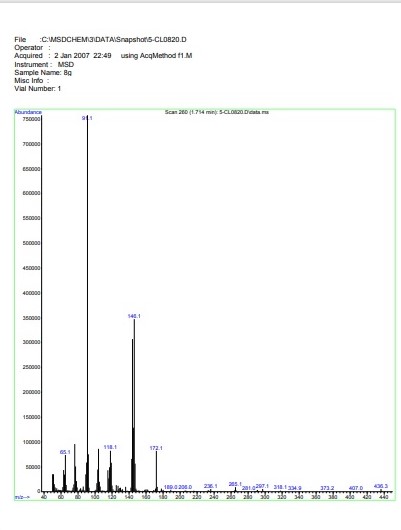 |
| --- | --- |
| 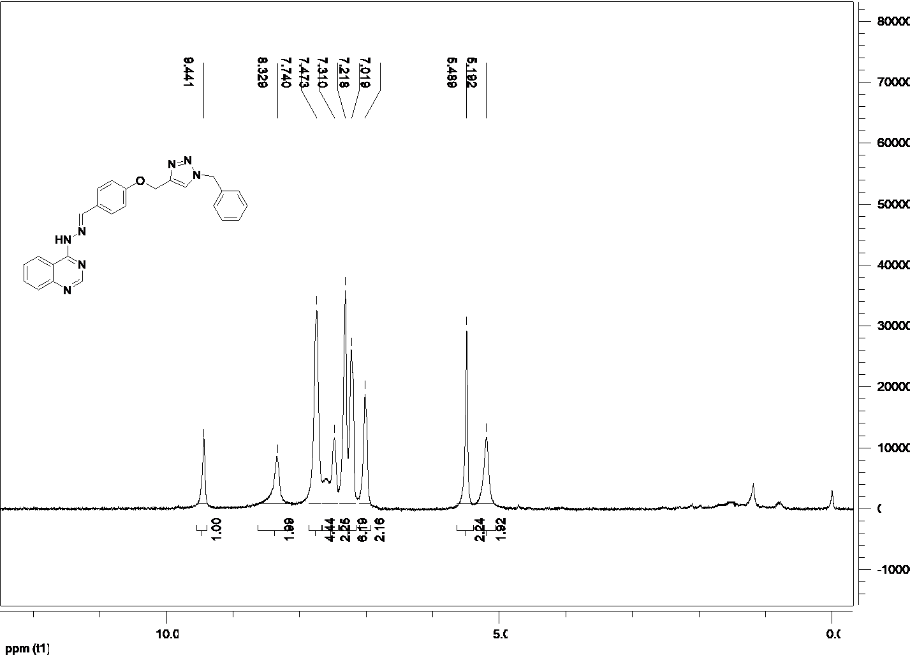 | 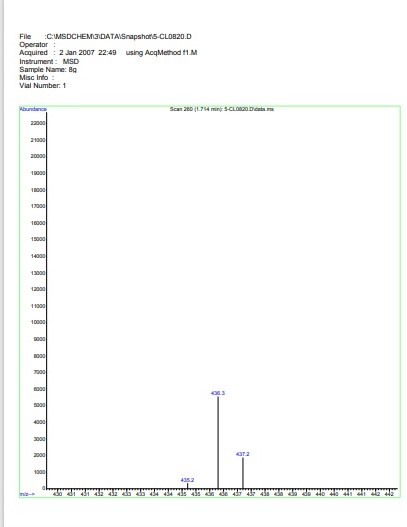 |
| **Supplementary Figure 8.** Chemical characterization of compound **8g.** | |

| 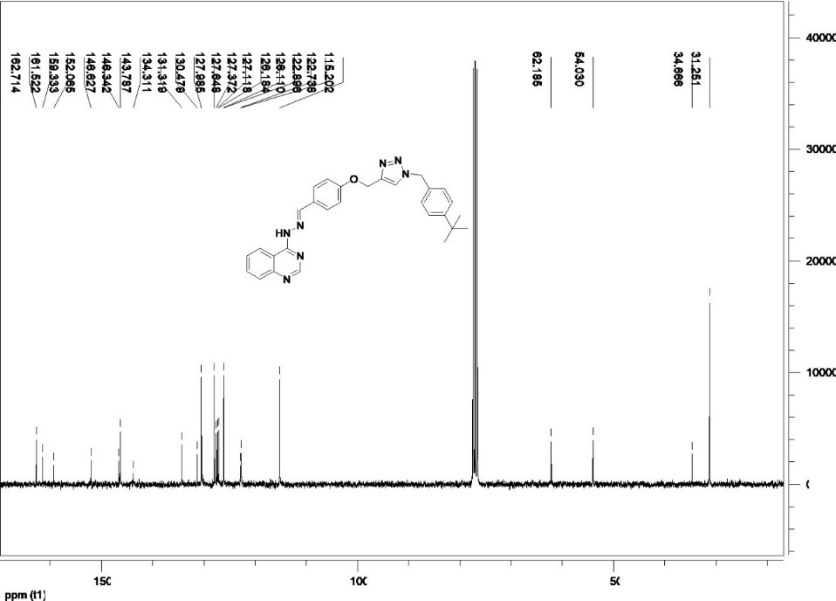 | 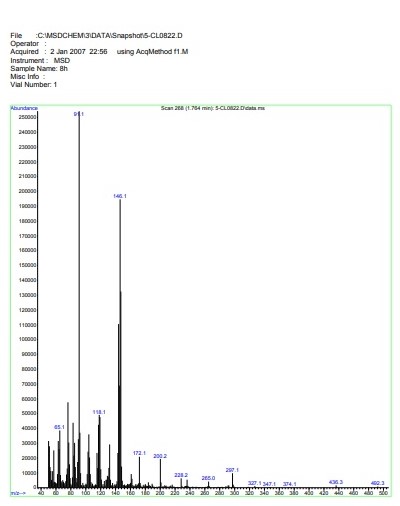 |
| --- | --- |
| 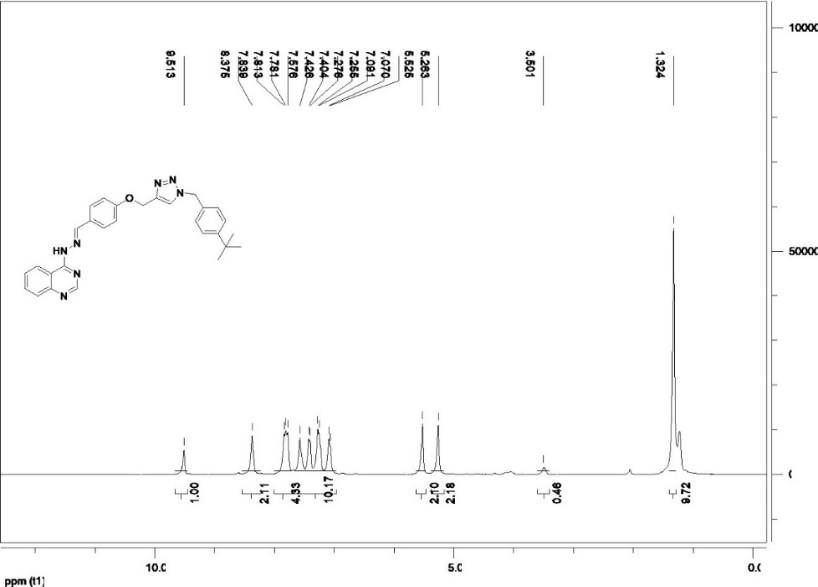 | 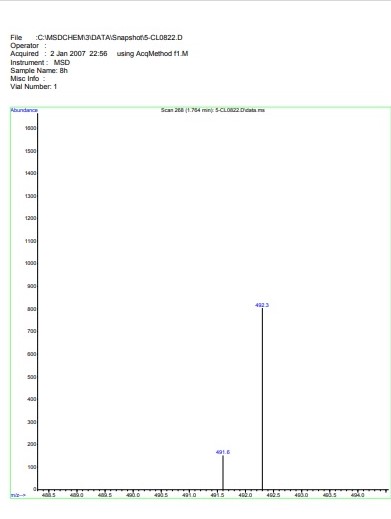 |
| **Supplementary Figure 9.** Chemical characterization of compound **8h.** | |

| 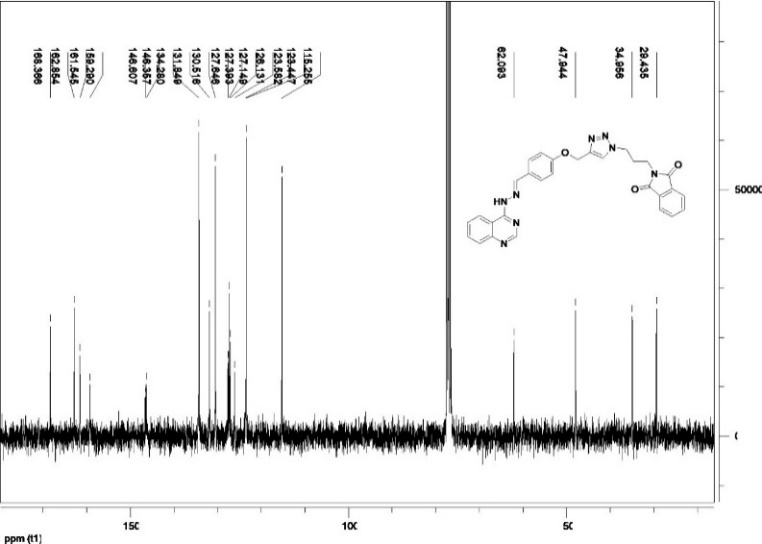 |
| --- |
| 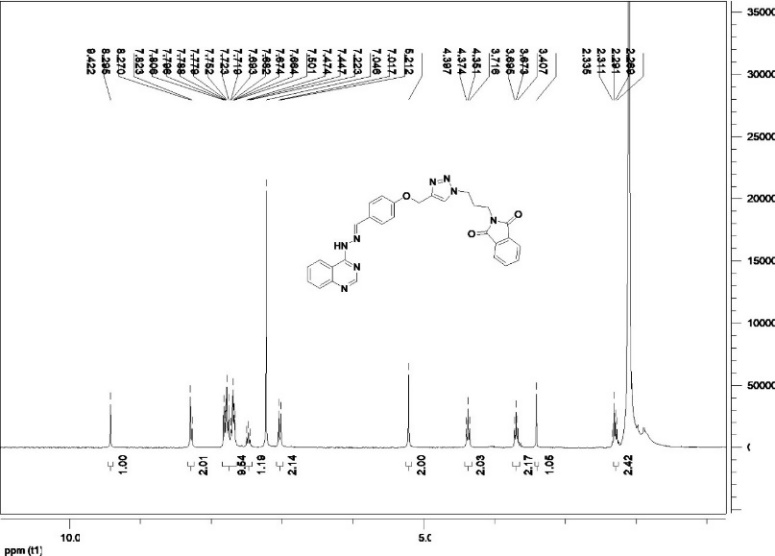 |
| **Supplementary Figure 10.** Chemical characterization of compound **8i.** |

.
